# Guidelines for mechanistic modeling and analysis in cardiovascular research

**DOI:** 10.1152/ajpheart.00766.2023

**Published:** 2024-06-21

**Authors:** Mitchel J. Colebank, Pim A. Oomen, Colleen M. Witzenburg, Anna Grosberg, Daniel A. Beard, Dirk Husmeier, Mette S. Olufsen, Naomi C. Chesler

**Affiliations:** ^1^Edwards Lifesciences Foundation Cardiovascular Innovation and Research Center, Department of Biomedical Engineering, University of California, Irvine, Irvine, California, United States; ^2^Department of Biomedical Engineering, University of Wisconsin-Madison, Madison, Wisconsin, United States; ^3^Department of Molecular and Integrative Physiology, University of Michigan, Ann Arbor, Michigan, United States; ^4^School of Mathematics and Statistics, University of Glasgow, Glasgow, United Kingdom; ^5^Department of Mathematics, North Carolina State University, Raleigh, North Carolina, United States

**Keywords:** computational modeling, mathematical model, model calibration, quantitative biology, simulation

## Abstract

Computational, or in silico, models are an effective, noninvasive tool for investigating cardiovascular function. These models can be used in the analysis of experimental and clinical data to identify possible mechanisms of (ab)normal cardiovascular physiology. Recent advances in computing power and data management have led to innovative and complex modeling frameworks that simulate cardiovascular function across multiple scales. While commonly used in multiple disciplines, there is a lack of concise guidelines for the implementation of computer models in cardiovascular research. In line with recent calls for more reproducible research, it is imperative that scientists adhere to credible practices when developing and applying computational models to their research. The goal of this manuscript is to provide a consensus document that identifies best practices for in silico computational modeling in cardiovascular research. These guidelines provide the necessary methods for mechanistic model development, model analysis, and formal model calibration using fundamentals from statistics. We outline rigorous practices for computational, mechanistic modeling in cardiovascular research and discuss its synergistic value to experimental and clinical data.

## INTRODUCTION

Computation is an integrative component of today’s scientific research process. Engineering, physics, and climate science have successfully employed computational modeling in scientific research and, more recently, in regulation and predicting future events. Modeling and simulation are also important tools in biomedical discovery and are now a regulated practice for medical device design within the Food and Drug Administration (FDA) (1, 2). Mechanistic, in silico models have had notable success in representing physiological mechanisms through mathematical relationships, especially in the case of cardiovascular research. Modeling has traction in multiple other physiological domains, including drug discovery and innovation (3) and advancing medical device technologies (1). However, as with other research techniques, modeling and simulation should be reproducible and credible, requiring rules, guidelines, and best practices.

**Figure 1.**
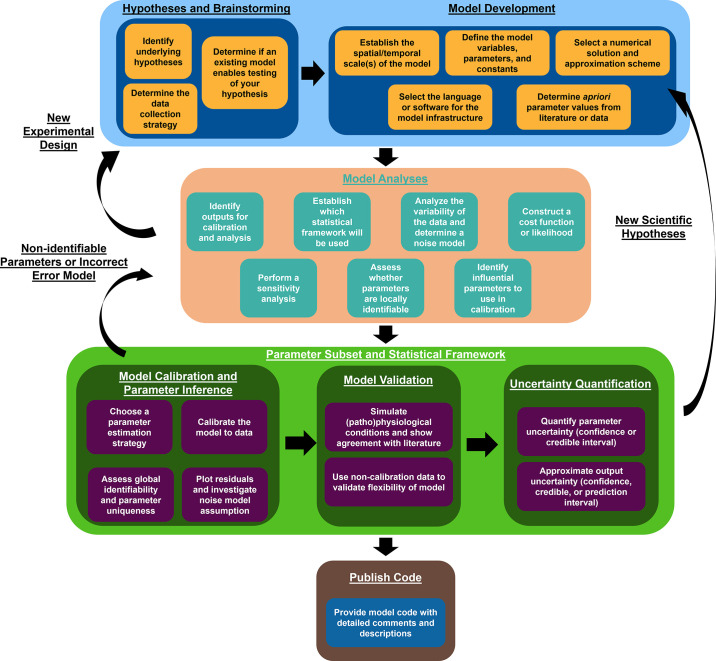
Proposed workflow for implementing and using computational models. The sections of the manuscript follow the workflow outlined here.

The goal of this guidelines article is to establish a framework for in silico computational analyses as a tool for cardiovascular research, as outlined in Fig. 1 and Table 1. Published recommendations for credible modeling and simulation practices (1) form the foundation of this article, and we highlight these practices in the context of cardiovascular research. We review the importance of modeling and simulation, provide introductory descriptions of several mathematical and statistical modeling techniques, identify the use of modeling in combination with experimental and clinical data, and establish a set of guidelines for in silico cardiovascular analyses suitable for the *American Journal of Physiology-Heart and Circulatory Physiology* (*AJP-Heart and Circ*).

**Table 1.** Proposed guidelines for using computational models as a tool for understanding cardiovascular function

Analysis Step	Minimum Requirement	Additional Analyses	Examples
Hypothesis step	- Identify how a computational model will help test hypotheses. - Brainstorm possible model outputs.		
Model development	- Investigate if there are existing models that are relevant. - Identify experimental outputs. - Establish model variables and parameters with their units. - Quantify model parameters using expermental data or literature.	- Perform a dimensional analysis to identify redundant model components. - Compare numerical schemes. - Perform mesh or discretization error analyses.	(4) (3, 5–8)
Model analyses	- Specify which model outputs are of interest. - Select a statistical framework. Construct an error metric for calibration. - Perform an informal “sensitivity analysis” changing one factor at a time. - Select parameters that are influential on the outputs needed for model calibration.	- Formal local sensitivity analysis. - Assess measurement error model. - Perform local parameter identifiability. - Global sensitivity analysis. - Calculate the profile likelihood using synthetic data. - Reduce model complexity.	(9, 10) (11, 12) (13–15) (9, 16) (5)
Model calibration	- Identify a frequentist or Bayesian inference method. - Conduct parameter estimation and assess accuracy to measured data. - Determine whether more data are necessary.	- Perform a multistart optimization and test for global identifiability. - Investigate residual error and assess noise model assumption. - Use additional synthetic data to see if model fitting is improved. - Calculate information criteria and select the best model.	(10, 17–20) (11, 12, 21) (12, 22, 23)
Uncertainty quantification	- Calculate parameter confidence or credible intervals. - Approximate the model output uncertainty via “informal” sensitivity analysis.	- Use sensitivity matrix to calculate asymptotic confidence and prediction intervals. - Determine output uncertainty using sampling or asymptotic frequentist analyses.	(10, 24) (17, 25–27)
Code publishing	- Create a folder available for reviewers and readers upon request. - Comment and document code to link manuscript details to functions and equations.	- Upload code to a public repository. - Upload nonidentifiable data to repository for additional analyses. - Provide a step-by-step instruction document for running the model.	(28, 29)

## A BRIEF REVIEW OF IN SILICO MODELS

In silico models span multiple spatiotemporal scales and can incorporate both physics-based mechanisms and phenomenological behavior as evidenced by experiments. For example, cardiovascular mechanics at the tissue and organ level are typically simulated through fluid and solid mechanics models. In contrast, many electrophysiological, cell, and systems-level models incorporate mathematical relationships from other domains (e.g., chemical kinetics and electrical circuit theory). Here, we briefly summarize several types of in silico mechanistic models that have been used in understanding (ab)normal cardiovascular function. We begin with classical models of solids and fluids and then move toward biology-driven and systems-level cardiovascular models. These models were selected because they are foundational studies in computational physiology, some of which are from *AJP-Heart and Circ*, that bridge cardiovascular physiology to the computational domain. This is by no means a complete catalog of the numerous works in the field, and readers are encouraged to seek out other reviews on specific modeling topics (30–35) to supplement this guidelines article. Our guidelines illustrate a robust methodology that is more general than the examples given.

### Solid Mechanics Models

Solid mechanics models integrate concepts from continuum mechanics to simulate deformations and stresses in cardiac and vascular structures. They are typically represented using partial differential equations (PDEs), which account for dynamic changes in space and time. Solid mechanics models can be constructed from patient imaging data to simulate patient-specific cardiac or vascular deformations under load. These models typically use a three-dimensional (3D) finite element method framework to simulate cardiac chambers’ (36–38), valves’ (34, 39, 40), and/or vessels’ structural mechanics (41). For example, Finsberg and colleagues (38) recently constructed an image-based finite element model of the left and right ventricles (LVs and RVs, respectively) informed by patient-specific cardiac magnetic resonance (CMR) imaging. Their model simulations of dynamics in control and pulmonary arterial hypertensive conditions matched well to patient-specific pressure-volume loop data. Pfaller et al. (42) combined 3D MRI images from all four heart chambers, adipose tissue, and great vessels in a finite element model of pericardial-myocardial interactions. The authors simulated pressure-volume loops and showed excellent agreement to measured LV volume and wall displacements and provided in silico evidence that including pericardial constraints enable more accurate predictions.

Regarding vascular dynamics, numerous authors have used solid mechanics to simulate stress-strain relationships in idealized, thick-walled cylinders representing vasculature (43–46). The seminal work of Holzapfel et al. (46) described the complex, multilayer behavior of large arteries using solid mechanics principles. Their model included medial and adventitial constituents, fiber direction, and residual stress, all contributing to model outputs that parallel experimental data from ex vivo rabbit carotid arteries. Others, like Ramachandra and Humphrey (47), have implemented two-dimensional (2D) models of vascular mechanics that consider average circumferential and axial stress in arteries. The authors illustrated differences in dimensions between the left and right mouse pulmonary arteries but similarities in the stress-strain behavior as measured experimentally and predicted from their model.

Computational solid mechanics models are also gaining traction in the medical device industry. Valve annuloplasty and artificial heart valves are commonly used to help correct cardiovascular abnormalities, such as mitral valve regurgitation, but are now being leveraged with computation. For example, Wong et al. (48) investigated the shape of mitral annuloplasty rings using finite element simulations and concluded that the shapes of both saddle-shape and asymmetric annuloplasty rings led to similar predictions of LV fiber stress and valve leaflet curvature. Oomen et al. (49) combined a finite element model with ex vivo biaxial testing data from human aortic and pulmonary valves. The authors showed that circumferential stresses were significantly different across valve types and that, more importantly, differences in functionality and valve stress between fetal and adult heart valves likely attributed to age-dependent collagen remodeling.

### Fluid Mechanics Models

Simulating spatially varying blood flow in a vessel or heart chamber requires computational fluid dynamics (CFD) models. Most of these models rely on numerical solutions to the Navier-Stokes equations in combination with mass or energy conservation equations and can simulate blood flow within heart chambers or in blood vessels. For instance, Zambrano et al. (50) constructed a 3D CFD model using medical imaging data to study hemodynamics in pulmonary arterial hypertension. The authors simulated pressure-displacement dynamics that matched data from cine magnetic resonance images in the main pulmonary artery. Three-dimensional CFD models typically assume rigid cardiac or vascular walls and ignore the dynamics of wall motion. Combining CFD and solid mechanics leads to fluid-structure interaction (FSI), where CFD simulations are coupled to a moving, deformable structure. The seminal 1955 paper by Womersley (51) provided the theory for oscillatory flow in an elastic tube, a first approach to FSI that also provided insight into how viscous and inertial forces shape the velocity profile of flowing blood. A major FSI approach is the immersed boundary method, developed by Charles Peskin in the 1970s (17, 52), which can simulate blood flow around moving heart valves and in deformable ventricles. Another FSI approach used by Taylor et al. (53) is the arbitrary Langrangian-Eulerian (ALE) framework, which simulated 3D blood flow in deformable vessels. The work by Taylor et al. led to the coupled momentum method by Figueroa et al. (54), which is the 3D-FSI methodology used in both the SimVascular (55) and the CardiovasculaR Integrated Modeling and SimulatiON (CRIMSON) (56) packages.

While 3D-FSI models are often considered the gold standard for CFD modeling, there are reduced-order models that can also describe blood flow and vascular wall deformations. The 2D CFD model developed by Ghigo et al. (57) accounted for cylindrical blood flow dynamics in the axial and radial direction and provided physiological predictions of energy losses across stenoses. The one-dimensional (1D) CFD framework is another common alternative to 3D vascular blood flow models. Whereas the former 3D model provides insight into spatially varying flow patterns within a blood vessel, 1D models assume axisymmetric axial blood flow. This enables a more efficient model that can be solved in a network of vessels, such as the entire coronary, cerebral, or pulmonary circulations, to understand pulse wave propagation (35, 58). Several studies have found that 1D and 3D models provide similar simulated outputs. The study by Xiao et al. (59) compared 1D simulations in the ascending and descending aorta to 3D-FSI results and found that pressure-flow relationships were nearly identical. Blanco et al. (60) showed that simulated fractional flow reserve (FFR) in the coronary circulation was nearly identical between a rigid walled 3D CFD model and its compliant, 1D counterpart. The use of physiological-driven boundary conditions, such as the structured tree model by Olufsen et al. (58), enables simulations in blood vessels that cannot be captured by traditional imaging modalities. In general, the quantities of interest for the problem (e.g., proximal wall shear stress or wave reflections due to distal vascular disease) should dictate which CFD model type is used.

### Cardiac Muscle Mechanics Models

Mathematical models of cross-bridge dynamics can simulate the interactions between actin and myosin filaments, and parallel concepts of the sliding filament theory. In contrast to most solid mechanics models that focus on stress-strain behavior at the macroscopic level, models of cardiac muscle are usually on the microscopic scale. Individual sarcomeres, on the order of 2 μm, can be simulated in response to changes in the local biochemistry, typically with calcium concentration changes. The work by Hill (61) and later Huxley (62) developed what is now a fundamental model of cross-bridge cycling, described in detail by Niederer et al. (63). Multiple innovations on Huxley’s model have been developed, including models with multiple binding steps between actin and myosin heads (64–68) and frameworks that account for cellular energetics (64, 69). These models share similar features to Huxley’s original work (e.g., length-dependent force generation) but also include calcium binding and calcium sensitivity, active force mechanisms, and passive stiffness due to molecular components such as titin (67, 68). Most cross-bridge models treat muscle fibers as a motorized system, with active force generation driven by changes in intracellular calcium and stretch (e.g., the Frank-Starling relationship). Similar methodologies have also been applied to understanding vascular smooth muscle function (70–72), although the underlying mechanisms of force generation are different between cardiac and smooth muscle. The recent review by Niederer et al. (63) outlines key studies that innovated on the original Hill and Huxley models, as well as the future avenues for the use of cardiac muscle models in understanding complex cardiac pathologies.

### Cell Signaling Models

Cell function is typically described through kinetic, logical, or phenomenological relationships. The former two methods are the most common for developing cell signaling network models (73). Kinetic models are typically the mechanistic model of choice when simulating time-dependent cardiac and vascular cell function over seconds to hours. Examples published in *AJP-Heart and Circ* include models of vascular endothelial growth factor (VEGF) binding in endothelial cells (18), mitochondrial interaction with calcium handling in cardiomyocytes (69), and nitric oxide signaling between vascular endothelial and smooth muscle cells (71). In contrast, logic-based network models simulate each component of a system using Boolean operations, i.e., using “OR,” “AND,” and “NOT” statements. These model systems can describe up- and downregulation of genes, proteins, and other biological motifs using qualitative information or data (74) and are commonly used to analyze gene regulatory networks (75). Both cell models have had marked success in the pharmaceutical industry (76, 77) and are considered a key component of quantitative systems pharmacology. The work by Nelson et al. (78) used logic-based network modeling of cardiac fibroblast signaling in combination with machine learning to identify possible targets for maladaptive cardiac fibrosis. The authors found that Src inhibition through a phosphatidylinositol 3-kinase-dependent signaling cascade was responsible for actin-myosin stress fiber formation and procollagen I production, providing a pharmaceutical target for cardiac fibrosis. These models are becoming more valuable as technologies for acquiring data improve at the cell level and smaller, especially at the genomic level, as discussed next.

### Metabolism and Genomics Level Models

Cell metabolism is critical for cardiovascular function, especially in contractile cells such as cardiomyocytes and vascular smooth muscle cells. Computational models of cell metabolism have been developed by several groups, including those focused on metabolism in cardiovascular-specific cell types. The work by Zhou et al. (79) developed a mechanistic model of mitochondrial respiration to compare to experimental data in isolated perfused rat hearts. The authors used the model to predict glycogen breakdown and lactate production during ischemia and argued that glycolysis is localized to a subdomain of the cellular cytosol. Bazil et al. (80) developed a kinetic and thermodynamic framework for mitochondrial energetics, ATP synthesis, and reactive oxygen species (ROS) generation that predicted similar trends in oxygen rate consumption as experimental data from rat hearts ex vivo.

In recent years there has been an exponential increase in the technologies available for sequencing and high-throughput ‘omics analyses (e.g., proteomics, transcriptomics, genomics). Data from ‘omics investigations are typically analyzed using data-driven and statistical approaches, e.g., using principal component analysis (PCA), t-distributed stochastic neighbor embedding (t-SNE), or uniform manifold approximation and projection for dimension reduction (UMAP) (81–83). Several groups have also used semimechanistic or fully mechanistic models to understand high-dimensional relationships that are a byproduct of ‘omics data. For instance, Jin et al. (84) devised the “CellChat” framework, which provides information about cell-to-cell communication via cell ligand and receptor expression from single-cell RNA-sequencing (scRNA-seq) data. Cang and Nie (85) developed a computational infrastructure for connecting individual cells across different measurements by “spatially optimal transporting the single cells” (SpaOTsc). In contrast, the work by Lai et al. (86) sought to describe the role of microRNA (miRNA) signaling in gene regulatory networks using more fundamental promotor/inhibitor signaling models. The use of models in the context of metabolomics, proteomics, and genomics is a rapidly expanding area that will likely adapt to cutting-edge sequencing technologies.

### Electrophysiological Models

Models of cardiac electrophysiology combine kinetic models of action potential development with spatial domains representing cardiac tissue. As detailed more explicitly in the reviews by Carusi et al. (30) and Clayton et al. (87), the simulation of action potentials can be strictly time dependent or have 1D, 2D, or 3D spatial frameworks. Electrophysiological models with 3D domains are often separated into bidomain and monodomain models; the former account for differences in intracellular and extracellular conductivities, while the latter simplify this assumption and apply a uniform conductivity across the domain (87). Several researchers have used this model format to simulate both physiological and pathological cardiac electrophysiology as seen in clinical data (30, 87, 88). The study by Prakosa et al. (89) simulated 3D ventricular electrophysiology in both swine and human geometries after myocardial infarction. The authors computed optimal ventricular radiofrequency ablation treatments in both cohorts and showed that the patient-specific predictions of cardiac activation after targeted ablation agreed with clinical data. The follow-up study by Boyle et al. (90) used a similar modeling framework to simulate targeted ablation of persistent atrial fibrillation. Again, the authors showed how patient-specific electrophysiological models and simulated treatment could identify optimal ablation targets that led to successful clinical procedures. A promising global initiative using cell signaling models of cardiomyocyte behavior is the Comprehensive In Vitro Proarrhythmia Assay (CiPA) initiative (91). The initiative is supported by several organizations, including the FDA and uses mechanistic cardiomyocyte models to predict the risk of drug-induced effects and arrhythmias, expediting clinical trials (9).

### Models of Cardiovascular Growth and Remodeling

A more recent area of computational research has been simulating the temporal evolution of cardiac (92–95) and vascular (96–98) growth and remodeling. In this framework, model geometry and parameters adapt when a mechanical (e.g., stretch or stress) or biochemical [e.g., neutrophil release from matrix metalloproteinases, matrix metalloproteinase, after myocardial infarction (99)] stimuli are altered and deviate from their homeostatic set point. The goals of these models are to predict constituent and geometric changes in cardiovascular structures due to acute (e.g., myocardial infarction) or chronic (e.g., systemic hypertension) conditions.

Two theoretical approaches are used to model growth in response to altered mechanical stimuli: kinematic growth theory and constrained mixture theory. In the kinematic growth framework, first formalized by Rodriguez et al. (100), the total deformation gradient tensor is multiplicatively decomposed into an elastic and growth component. A “growth rule” is formulated to relate a change in mass of a mechanical constituent to a change in the growth deformation gradient tensor. The advantages of the kinematic growth theory include its relative simplicity and low computational cost. It has been primarily used to model cardiac growth. For example, Witzenburg and Holmes (94) compared eight different LV growth laws under simulated pressure and volume overload. Their results showed that a select few of the growth laws could reproduce the time-dependent wall thickening and/or dilatation found in various animal models of pressure or volume overload, respectively, in the literature.

In contrast, the constrained mixture method is more complex and simulates the change in mass of multiple tissue constituents simultaneously while accounting for their unique material properties and reference states (97). This approach is often used to describe vascular growth and remodeling for tissue constituents such as collagen, elastin, and vascular smooth muscle cells. The constrained mixture method established and made popular by Humphrey (97) has had success in capturing the time-dependent remodeling of the large arteries and has been recently used with mechanotransduction and cell signaling pathways to understand aortic vascular adaptation (101). In contrast to the kinematic growth theory, it is more computationally expensive since it requires tracking of a multitude of reference configurations for each tissue constituent. A homogenized version of the constrained mixture theory has recently been introduced to reduce this high computational cost (24, 102).

### Systems-Level, Lumped Parameter, and Multiscale Models

Different components of the cardiovascular system across multiple spatial scales are affected in disease. Multiscale systems-level models can be developed to address this by combining different model frameworks. To do this, lumped parameter cardiovascular models, sometimes called reduced order or zero-dimensional (0D) models, are commonly used.

The Windkessel model is one of the first and most widely used lumped parameter models, in which hemodynamic concepts are paralleled to circuit theory. This methodology uses Ohm’s law, resistance, and capacitance theory, and concepts of impedance to quantitatively describe features of the cardiovascular system (103, 104). The study by Olufsen and Nadim (105) derives these lumped parameter representations from the 1D hemodynamic system and shows how assumptions on the model domain (e.g., small radii) can enable their use in describing cardiovascular dynamics. Lumped parameter models are often combined with simplified models of cardiac function, like elastance models or “single fiber” models of active and passive force generation (106, 107). These models can also be integrated with other model frameworks. For example, Fan et al. (19) developed a reduced order systems model that included the LV, a lumped parameter systemic circulation, and a model of flow regulation and perfusion in the coronary circulation. The authors simulated coronary flow dynamics that paralleled experimental findings in individuals with mechanical dyssynchrony.

Models of other physiological processes can also be integrated in a multisystems-level framework. For instance, Arthurs et al. (108) combined a lumped parameter model of coronary blood flow with a metabolic and adrenergic control model to simulate myocardial oxygen supply during exercise. The authors showed that exercise reduced coronary vascular resistance and elevated myocardial oxygen availability. Geddes et al. (25) combined a lumped parameter model of the LV and systemic circulation to a baroreflex control module and simulated the effects of blood pooling during in the lower body during head-up tilt. Their results provided insights into potential mechanisms of postural orthostatic tachycardia syndrome.

Finally, many of these modeling approaches have been combined into multiscale models, which account for different spatial or temporal scales. These models incorporate different components of the cardiac or vascular tissue (e.g., cardiomyocyte action potentials, sarcomere shortening, and ventricular contraction) to simulate function (13, 45, 64, 66, 109). Typically, several single-scale models (e.g., lumped parameter, cross-bridge kinetics, and cell signaling) are linked to represent the cardiovascular tissue across different spatial scales. The work by Arts et al. (110) and later Lumens et al. (5) developed the CircAdapt and three-segment (TriSeg) heart model, respectively, which incorporate cardiac and vascular adaptation rules, systems-level models of the circulation, biventricular interaction, and single-fiber models of sarcomere force development. Lopez et al. (66) and Marzban et al. (64) developed a multiscale model of ventricular energetics that included mitochondrial function, a multistate cross-bridge model, biventricular interaction, and a lumped parameter model the circulation. Their model provided evidence that altered ATP and inorganic phosphate levels impair cross-bridge cycling and overall ventricular function.

In summary, computational models of cardiovascular physiology can simulate (ab)normal human physiology with varying complexity and across multiple scales. Each model framework can be combined with experimental data to help test underlying hypotheses. In the next sections, we describe methods for developing, calibrating, and evaluating these computational models. These guidelines can be applied to any cardiovascular model and help ensure that model analyses are robust, reproducible, and quantifiable in cardiovascular research.

## MODEL DEVELOPMENT

We provide a detailed, step-by-step guide for developing and adapting model frameworks to use within *AJP-Heart and Circ.* These guidelines also extend to any study that uses existing or novel simulation platforms in cardiovascular science. We specify terminology for components of the model framework and walk through the model development pipeline. Similar criteria and guidelines for model development and use can be found in the American Society for Mechanical Engineering (ASME) verification and validation (V&V) 40, which focuses on modeling and simulation related to medical devices (14).

### Computational Models as Vehicles to Test and Refine Hypotheses

Computational models and their simulations can be derived from existing or novel hypotheses about a biological system. A computational, mechanistic model may be constructed to formally encode an underlying hypothesis about how a system operates (111). Hence, a mathematical representation of a biological or cardiovascular system is, by design, a hypothesis that can be tested with experimental data and measurements. The choice of model should enhance the experimental design, and, vice versa, the experiments should help inform the model. For instance, clinical arterial blood pressure and heart rate data should be combined with a model that at least includes model components for these measurements. As discussed throughout, the data will guide how a model is used (e.g., calibration or validation) to help test or investigate a hypothesis. Conversely, the model may guide future experiments or measurements themselves. If a model has a specific mechanistic component that has not been studied extensively, then the model may inform a new set of experiments or hypotheses to test. In the above example, a model may simulate arterial blood pressure and heart rate, but the explicit relationships between these two variables may drive a new experiment to test whether a mechanism (e.g., the baroreflex) is represented correctly by the model. Sensitivity analyses, discussed later, may also provide insight into which parameters have an impact and drive new measurements that inform these parameters. Thus the development and refinement of hypotheses using mechanistic models are necessary ways to link computation and experimental or clinical measurements and can expedite new hypothesis generation.

### Types of Computational Models

We distinguish mechanistic models from data-driven models (10). Mechanistic models rely on mathematical relationships constructed from underlying physics or biological hypotheses that may or may not have observations. For example, consider the biochemical system governed by:

(*1*) $$A+B\begin{array}{c} k_{2}\\ ⇄\\ k_{1} \end{array}C,$$

where *A* and *B* are two compounds that can follow a forward reaction to make the product, C, at rate *k*_1_. The product will also degrade to the compounds with some rate *k*_2_. This system can be described mechanistically using differential equations and laws of mass action kinetics (15). For instance, changes in *C* can be described by:

(*2*) $$\frac{\text{d}}{\text{d}t}\left[C\right]=k_{1}\left[A\right]\left[B\right]-k_{2}\left[C\right].$$

The above is read as “*C* increases at a rate proportional to the amounts of the compounds, *k*_1_[*A*][*B*], and decreases at a rate proportional to the current amount of *C* available, *k*_2_[*C*].” Other examples include the fluid and solid mechanics models, cross-bridge kinetics, and lumped parameter models discussed earlier. These models explicitly represent the hypothesized mechanisms of the system in mathematical formulas.

In contrast, data-driven models, including artificial intelligence (AI) as well as statistical and machine learning models, rely on observables only. These models relate covariates (i.e., the abscissa) to a response variable (the ordinate) or, in the case of classification, to a specific grouping. Some examples include linear and nonlinear regression models, Gaussian processes, *k-*means clustering, decision trees, and neural networks. The goal of data-driven models is typically to infer correlations between observables and (if applicable) responses and construct an accurate predictive tool for new observations. Statistical models can draw conclusions on whether relationships between data are significant (e.g., ANOVA) under a priori assumptions about the sampling or probability distribution of the data. As noted in several perspective articles (10, 112), mechanistic and data-driven models are useful in different circumstances or can be combined to understand cardiovascular disease (113). In this guidelines article, we focus on the analysis and use of mechanistic models.

The type of mechanistic model should also reflect the level of detail necessary for scrutinizing the cardiovascular hypothesis. Scientific questions regarding subsystems or processes that are strictly time dependent, without any explicit spatial variability, can be analyzed using algebraic or ordinary differential equation (ODE) models. Systems of ODEs can be readily solved and analyzed in computing environments like MATLAB and Python. For data or hypotheses with both temporal and spatial variability, multicomponent ODE models or PDE models can be used. PDE models, especially those using the finite element method, can be computationally expensive to solve and require more specialized software (identified later in the text).

Another special class of models is stochastic differential equation models (SDEs). These models incorporate randomness (stochasticity) explicitly in the systems of equations and are widely used in finance but less often in physiology. Finally, agent-based models are gaining traction in biological applications (114). Agent-based models simulate spatiotemporal dynamics by allowing individual “agents” to respond to stimuli and/or their nearest neighbors. The work by Keshavarzian et al. (115), e.g., used an agent-based model approach to simulate vascular adaptation in response to endothelial cell, vascular smooth muscle cell, and fibroblast signaling, as well as various biochemical constituents. Agent-based models of cardiac adaptation have also been developed; Rouillard and Holmes (116) used agent-based modeling coupled with a finite element model of the LV to quantify region-specific collagen fiber response and scar formation after a myocardial infarction. Agent-based models are used more and more in the cardiovascular remodeling space and will likely provide new insight into the spatial heterogeneity in cellular response to chronic or acute injury (117).

In summary, mechanistic models can vary in spatial and temporal resolution and can be deterministic or stochastic. The model selected for a particular application or hypothesis should at least simulate the spatial and/or temporal resolution of the underlying cardiovascular function or measured data.

### Know the Difference: States, Parameters, and Constants

Mechanistic models consist of multiple variables, which can represent different quantities and components of the underlying mathematical equations. Fundamentally, a system will have independent and dependent variables. Independent variables are prescribed or updated (i.e., the ordinate of the system), whereas dependent variables evolve or change as the independent variable is incremented (i.e., the abscissa of the system). For example, in a kinetic reaction system, time is an independent variable while concentrations or numbers of reacting species are dependent variables. In spatially explicit models, both time and space variables are independent variables.

The idea of a state variable is specific to mechanistic modeling, dynamical (i.e., time dependent) systems, and computational biology. The states of a system, the dependent variables, are the main physical quantities of scientific interest. These states evolve or change with time and/or space and new inputs. These dependent variables are described mathematically through linear or nonlinear contributions of other states, independent variables, parameters, or constants. As discussed in model calibration, parameters should be interpreted as quantities of the system that are determined by *1*) available data, or *2*) changes in the physical system (e.g., environmental or physiological conditions). Parameters are sometimes synonymous with constants, although constants may indicate fixed universal constants (e.g., π, Earth’s gravitational acceleration, or Avogadro’s number). We advise that those using in silico models distinguish between states, parameters, and constants, especially if considering parameter inference for model calibration.

### Identifying Units and Establishing Mechanistic Relationships

A physical quantity can be defined in terms of its dimensions (e.g., length, mass, time, temperature) and units (m, cm, in for length; kg and g for mass, etc.) or systems of units [Systeme Internationale (SI) for m-kg-s or CGS for cm-g-s]. Consistent and clear unit usage is important when developing in silico models. For example, cell-level models may include solute concentrations (in molarity), rate constants (s^−1^), and membrane voltage (V). Lumped parameter models of the heart and vasculature typically use volume (mL or L), pressure (mmHg or KPa), and time (in s), as well as unit combinations describing elastance (mmHg·mL^−1^), compliance (mL·mmHg^−1^), and resistance (mmHg·s·mL^−1^). This is increasingly important for model reproducibility and innovation, as computational models may be scaled from one organism to another, e.g., from human to rodent (26, 118). Techniques like nondimensionalization can be useful in ensuring models are scalable and in reducing the number of parameters in a model.

Mechanistic models translate theory into mathematical expressions and relationships. Such models encode universal physical and thermodynamic principles (e.g., conservation of mass, momentum balance, etc.) into mathematical representations. In contrast, biophysical principles are often harder to describe mathematically and necessitate a different approach to model building. Typically, scientists will either apply established physical laws to a biological system (mass conservation, law of mass action, etc.) or use experiments to inform phenomenological models. A notable example is the Hodgkin-Huxley model for cardiac conduction, which applied an established physical model to a biological system with phenomenological modifications (119). The authors used voltage-clamp experiments in the squid giant axon to measure the different currents of sodium and potassium fluxes. A system of four differential equations describing membrane potential, derived from electrical circuit theory, was then used in this seminal work. While the fundamentals of the model system stem from theoretical methods for simulating voltage and current, the ion rate channels in their model are phenomenological and based on observations from experiments. This idea is now a backbone for multiple models of cardiac action potential, including the initial work by Noble and colleagues (120). Other examples include using electrical circuit theory to describe pressure-flow relationships in Windkessel models (103) and the mathematical formulations of spring-dashpot systems to describe length-tension development (63) and arterial viscoelasticity (44). Regardless of whether the mechanistic model is novel or is a previously established system, we advise that authors explicitly describe the units attributed to their model variables and provide insight into the biophysical meaning of the equations and variables in the computational model.

### Impact of Animal Species and Disease Comorbidities

In vivo preclinical experiments are a cornerstone of cardiovascular research. Computational models that integrate in vivo preclinical data will describe the underlying physiology of the specified organisms (e.g., rodents or large animals). This distinction, although obvious, is crucial to keep in mind. For instance, Tewari et al. (121) developed a lumped parameter model to interrogate the progression of pulmonary arterial hypertension in mice. Male mice were assigned to either normal oxygen conditions or a combination of hypoxic environment and the selective VEGF inhibitor SUGEN. Thus the models and model parameters are specific to male mice under these conditions and may not be extrapolated to describe other experiments accurately, e.g., female hemodynamics in mice under a different pulmonary hypertension protocol, without modification. This is especially important when referring to the constants and parameters reported by other authors, as the units, scale, and values used in each study should be interpreted in the context of the animal model used.

In vivo experimental models typically target a stimulus or physiological process that generates a specific phenotype, yet many experimental stimuli for recapitulating a cardiovascular disease phenotype led to comorbidities. This amplifies the complexity of the problem from a modeling perspective since components or subcomponents of other organ systems may need to be incorporated to account for these comorbidities. A relevant example is the development of animal models of heart failure with preserved ejection fraction (HFpEF) (122). In contrast to heart failure with reduced ejection fraction (HFrEF) due to pathologies like myocardial ischemia, HFpEF is attributed to multiple coinciding insults, such as chronic kidney disease, obesity, chronic obstructive pulmonary disorder (COPD), and diabetes (123). Hence, a computational model of HFpEF should include (or at least acknowledge) the controlled stimuli and affected physiological system that leads to HFpEF progression.

The animal species, sex, genotype, age, and environmental stimuli are important factors to note when referencing or repurposing a previously published model. Moreover, computational models should account for the impaired physiological system as a whole, or minimally, the limitations in the computational framework should be acknowledged if it does not explicitly account for the other comorbidities in the animal model.

### Physiological versus Pathological Function

Experiments are typically split into a control, wild-type, or sham group and a disease or mutation group. A similar approach is ideal when using computational models to investigate disease mechanisms. For instance, the studies by Lopez et al. (66) and Marzban et al. (64) constructed a multiscale model of cardiac energetics (including mitochondrial function and cross-bridge cycling) and then used the model in combination with hemodynamic and metabolomic data from sham and transverse aortic constriction (TAC) rats. The authors showed that the metabolic state (e.g., reduced oxidative ATP synthesis) was a major determinant of cardiac function and that simulating a “rescue” in the metabolic state in TAC animals restored sham equivalent hemodynamics in the model predictions. Another study by Colunga et al. (124) used a lumped parameter cardiovascular model with data from patients with successful and unsuccessful heart transplants. Their approach showed that the model parameters corresponding to pulmonary arterial elastance, systemic arterial elastance, and systemic arterial resistance correlated with ventricular power output and posttransplant mortality. These studies exemplify the modeling of both physiological and pathological experimental data. Like wet-laboratory experiments, computational studies should also compare physiological and pathological function, especially when proposing mechanisms of disease progression or (ab)normal function.

### Back to the Laboratory

The ability to link multiple spatial and temporal scales (cell, tissue, organ, and organism) through in silico models enables scientists to link data sources that are typically difficult to combine. Mechanistic models can generate new hypotheses that require additional experiments and innovations in existing protocols. This idea of model-informed experimental design is used in the physical sciences (125) and is gaining traction in the life sciences (126, 127). Prior cardiovascular studies have used this concept to study RV function (128) and cardiomyocyte action potentials (20). Using in silico modeling as an experimental design tool can identify important outputs, measurements, and parameters before conducting costly experiments, expedite the design process, and inspire experimental innovation. As discussed later in model calibration, in silico predictions may also inspire new or additional measurements to perform model validation. This step, which occurs after the model has been calibrated to experimental data, identifies if model simulations agree with the behavior of the physiological system. Model simulations can then drive new experimental designs as a validation of the model or inspire an entirely new study, as described previously in *Computational Models as Vehicles to Test and Refine Hypotheses*.

## MODEL CALIBRATION

When selecting a cardiovascular model, scientists need to consider which components or outputs of the model drive their underlying hypotheses. For example, using a model to forecast or predict cardiovascular function, i.e., the forward problem, can help postulate new physiological hypotheses or corroborate previously established phenomena. For example, Sun et al. (129) constructed a comprehensive cardiovascular model including the baroreflex response, biventricular function, pericardial constraint, and intrathoracic pressure and simulated various diseases. The authors recapitulated hemodynamic waveform shapes found in systolic heart failure, mitral regurgitation, cardiac tamponade, and the Valsalva maneuver.

Scientists may instead use the model in parallel with measured data for calibration (also called parameter estimation or parameter inference). This is typically called the inverse problem, where the model parameters are inferred to improve the similarity between the measurements and the model output. In the context of cardiovascular modeling, this could include fitting stress-strain or pressure-area data from isolated vessel testing experiments (130), inferring Windkessel model parameters from pressure-flow data (131), or calibrating a model of heart rate variability, respiratory control, and hemodynamics to continuous patient data (132). These examples are distinct from the concept of validation, which assesses whether a model can reproduce experimental or clinical observations without explicitly inferring the parameters to match the data. Several analysis steps that determine which parameters should be estimated and how to infer these parameters in a robust manner should be pursued before calibration. Here, we outline some basic theory and recommendations for model analysis.

### Frequentist and Bayesian Statistics

Model calibration and the uncertainty associated with the measurements (and parameters) can be described through either frequentist or Bayesian statistics. To make the analysis clear for nonstatistical audiences, consider the regression problem:

(*3*) y=M(θ)+ε

where *y* is the dependent variable, θ are the model inputs (e. g., parameters), and ε is the measurement error. The model, *M*(θ), describes the input-output relationship of the system (e.g., a linear regression equation). We explain measurement errors in more detail later in the text in *Measurement Error and Model Discrepancy*. Given measured data, we can determine the value of $\theta =\hat{\theta }$ that gives the “best fit” to the data. The parameter estimate $\hat{\theta }$ is an estimate of the true parameter of the system, θ*, which is unknown.

**Figure 2.**
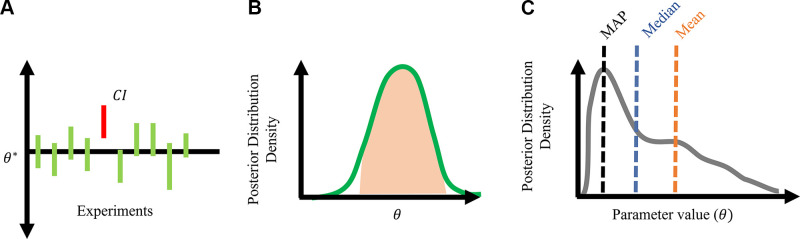
Statistical frameworks and terminology. *A*: confidence intervals in frequentist statistics. Note that the true value, θ^*, is fixed and 90% of experimental samples lead to a confidence interval (CI) containing the true value. One confidence interval (red) did not contain the true value, θ^*. *B*: credible interval under the Bayesian framework. The shaded region highlights the parameter values that are 90% probable. *C*: common terms used to describe probability density functions. The maximum a posteriori (MAP) estimate corresponds to the mode of the distribution, in contrast to the median and mean.

In the frequentist framework, we assume that the true parameters θ* have a fixed but unknown value and that the variability in estimates of $\hat{\theta }$ are purely an effect of measurement error. For example, differences in mean blood pressure across multiple measurements can be attributed to blood pressure cuff accuracy or intraoperator variability. Frequentist statistics assume that the data are a sample from a larger population and repeated experiments (new samples from the population) will give different values of $\hat{\theta }$ due to measurement error. The confidence interval around $\hat{\theta }$ measures the uncertainty in our estimate. In frequentist statistics, we describe a confidence interval using its significance level, e.g., 95%, with the interpretation “95% of repeated experiments will contain the true, fixed value of θ* in the constructed interval.” There is not a 95% probability that θ* is in the interval; it either is in the interval, or it is not. The confidence interval reflects uncertainty due to inherent sampling errors, which theoretically decreases as the quantity of data increases. A depiction of frequentist confidence intervals is provided in Fig. 2*A*, in which the red confidence interval denotes the sample population data that led to a confidence interval that did not contain the true parameter.

In contrast, the Bayesian framework assumes that the parameters of the system are random variables that follow a (typically unknown) probability distribution. Bayesian methods hinge on Bayes’ theorem, which relates the prior knowledge about the parameters (e.g., their upper and lower bounds or their mean value) and the likelihood of the data to construct a posterior distribution for the parameters. The prior distribution represents the uncertainty of the parameters before any data are used for calibration. For instance, if we are interested in determining the material properties of the human abdominal aorta using computational mechanics, then a prior distribution (e.g., a uniform distribution with equal probability across a range of values) on these parameters can be calculated from literature values (133). The likelihood is similar to a cost function or objective function and determines how likely the observed data are given the parameter values, model simulations, and knowledge of uncertainty in the system. Finally, the posterior distribution represents the updated belief about the parameters and their uncertainty using both the prior distribution and the likelihood of the data, given the model structure.

The benefit of the Bayesian framework is that the posterior distribution of a parameter can be interpreted more intuitively than the frequentist analog. A 95% credible interval, the Bayesian parallel of a confidence interval, is interpreted as “the parameter θ has a 95% probability of taking on values in the interval.” An example of posterior density is shown in Fig. 2*B*. One potential pitfall in using a posterior distribution is how to draw conclusions based on the shape and statistics of the distribution. When analyzing a posterior distribution, the maximum a posteriori value corresponds to the highest peak in the posterior density or the mode of the distribution. The is different from the mean or the median of the distribution, which is illustrated in Fig. 2*C*. The excellent review by Linden et al. (134) provides more details on Bayesian inference in the context of systems biology.

Most cardiovascular research uses the frequentist approach, where the parameters are treated as fixed, unknown values and the uncertainty is attributed to measurement errors. This is why statistical significance is tightly linked to the amount of data available, as larger samples lead to a better average representation of the true population. In the Bayesian framework, scientists impose their prior knowledge on how parameters are distributed and use their models and the likelihood to construct a posterior distribution. In contrast to classical statistics, Bayesian statistics treats parameters as random variables with their own intrinsic uncertainty, while also allowing for measurement error (135). The Bayesian methodology is increasingly used in several areas, including phylogenetics and big data analysis problems (136). Cardiovascular studies are also starting to use these methods more often (83, 124, 137–140). We later discuss the pros and cons of both methods from a numerical perspective but emphasize the importance of understanding statistical assumptions before applying them. This is especially important when returning to the underlying hypothesis and updating the experimental design. We recommend that the statistical framework for each study be acknowledged and that authors consider whether the frequentist or Bayesian framework is appropriate for their specific study. appendix a provides a more rigorous explanation of the Bayesian approach.

### A Priori Parameter Values

Physiological models should contain parameters that mimic or parallel biophysical quantities. As an example, the Michaelis-Menten model of enzyme kinetics describes the reaction rate and change of some enzymatic product, [*P*] (mol), at equilibrium as:

(*4*) $$\frac{\text{d}}{\text{d}t}\left[P\right]=V_{\text{max}}\frac{\left[S\right]}{K_{\text{S}}+\left[S\right]},$$

where [*S*] (mol) is the substrate, *K*_s_ (mol) is the Michaelis constant, and *V*_max_ (mol/s) is the maximum rate of product change (141). Since these parameters have a biophysical meaning, they can be informed from experimental data, literature, or some physiological knowledge. Importantly, they can be interpreted by a scientist in terms of biology. Another example includes the calculation of vascular resistance, which can be approximated by Poiseuille’s law:

(*5*) $$R=\frac{8\mu L}{\pi r^{4}}$$

with known blood viscosity μ (Pa s), blood vessel length *L* (cm), and blood vessel radius *r* (cm). Resistance plays a major role in most hemodynamic models (113, 142, 143), and can be used to relate pressure and flow. Models with multiple resistance elements can also be constructed from geometric data, where the physical dimensions of the vasculature can be used to calculate *Eq. 5*. The study by Marquis et al. (144) provides detailed insight into how a priori parameter values can be determined in larger, systems-level models.

In other cases, parameters may be indirectly related to a physiological variable. For instance, the study by Randall et al. (145) combined a model of cardiovascular function with a model of the baroreflex, which required identification of parameters linked to sympathetic and parasympathetic signaling. The authors used clinical and physiological insight to calibrate their model to data during a Valsalva maneuver. Randall et al. could then interpret these new parameters in the model as potential markers of autonomic dysfunction. As we discuss later, not all model parameters can be uniquely determined from data, requiring parameter “fixing.” This makes it especially important that parameters are identified and constructed based on current physiological understanding.

To understand disease progression, it is important to identify which parameters in the system are modified with pathology. If the model is being used to simulate pathology (e.g., changing a parameter that is linked to a biological hypothesis), then a model of physiological state is required beforehand. This often requires using published data or model frameworks to construct a baseline model. For instance, Kim et al. (146) devised a computational model of biventricular interaction that produced physiological pressure-volume dynamics in both heart chambers, especially during end-diastolic filling. After establishing the model, the authors showed that the model could replicate similar findings from patients with heart failure by changing model parameters hypothesized to affect heart failure progression.

We recommend that investigators clearly identify *1*) the biophysical interpretation of parameters, and *2*) the literature or experimental methods from which the parameter values are directly or indirectly based, including the ranges for parameter values.

### Quantifying Model-Data Agreement

One of the first steps in model calibration is identifying data for calibration. If all available data are used to calibrate the model, then conclusions can be drawn on the inferred parameters; however, model validation is limited. Alternatively, investigators may calibrate their model to only a subset of data and then compare simulations to the remaining validation data (discussed later) or predict behavior under different physiological or pathological conditions, such as exercise (22).

Regardless, care must be taken when identifying the objective of the calibration or “fitting” step. In the simple case of linear regression, the goal is to minimize the difference between the measured data and the regression model. Using our example in *Eq. 3*, we would try to find the parameters θ that minimize:

(*6*) $$J\left(\theta \right)=\sum_{i=1}^{N}{\left(y_{i}-M_{i}\left(\theta \right)\right)}^{2}$$

where *y* represents the data and *M*(θ) is the model simulations. In the above equation, *J* is the cost function, and *N* is the number of available data points for calibration. The above formulation is the ordinary least squares (OLS) solution, which is a classical problem in statistics.

The calibration of in silico cardiovascular models to clinical and experimental data in the literature is usually done in a more heuristic manner and deviates from the OLS approach. This is attributed to *1*) limited and noisy experimental data, *2*) complex, nonlinear computational models, and *3*) experimental data that vary in their measurement uncertainty. An example of the latter would be using clinical pressure data from catheterizations or noninvasive pressure estimates from echocardiography while also using cardiac output data with different magnitudes and units (120 mmHg vs. 5 L/min).

These calibration problems require reweighting and scaling the different data sources. Rather than using a heuristic approach, we recommend using more advanced (but robust) statistical techniques, like weighted least squares. For example, Olsen et al. (22) systematically match their lumped parameter model of the cardiovascular system to dynamic LV and RV volumes, aortic and pulmonary artery flow, and multiple systolic, diastolic, and mean pressure measurements from right heart catheterization. Using weighted least squares, the authors circumvented difficulties induced by differences in measurement units and magnitudes. The likelihood function used in Bayesian inference can also be adapted to handle differences in data magnitude and measurement uncertainty (see appendix b for more detail on likelihoods and cost functions).

Thus the structure of the cost function (or likelihood) will be problem dependent and will be driven by differences in both model composition and available data. We recommend that scientists report the components of their cost function or likelihood, as well as the available data, and that robust statistical frameworks be used for model calibration.

### Sensitivity Analyses

Sensitivity analysis quantifies how changes in the model parameters affect the simulated outputs. Scientists should quantify how influential parameters are on their model outputs. Determining “noninfluential” parameters can be difficult and somewhat heuristic, yet parameters that have minimal impact on the model outputs should be set to a fixed value. Identifying noninfluential parameters can simplify the model and decrease the number of free parameters. For instance, if all the parameters involved in a specific component of a model are noninfluential, it may be advantageous to remove that entire state of the system. This was the approach by Randall et al. (147), who used sensitivity analyses on established baroreflex models that included aortic, carotid, or a combination of the two baroreceptors to simulate heart rate fluctuations in response to the Valsalva maneuver. Parameter fixing or “subset selection” promotes unique model fits to experimental data and reduces the chance of having nonreproducible results (148).

**Figure 3.**
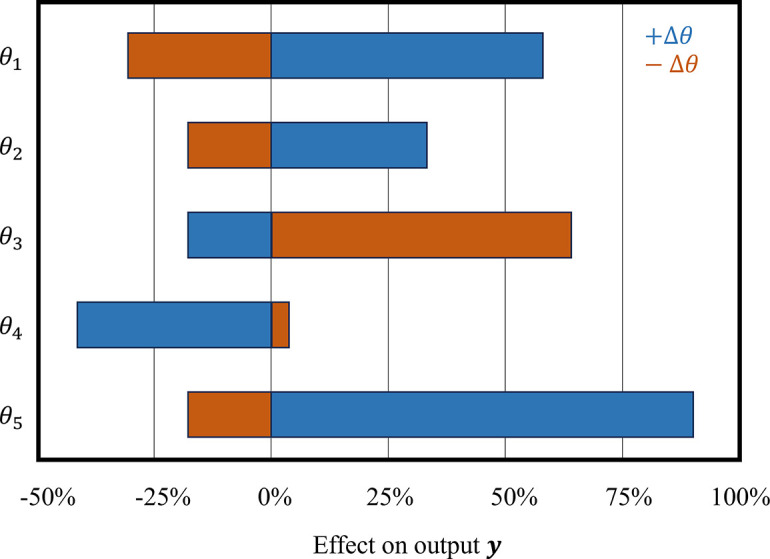
An example tornado plot. The effects of changing parameters (±Δθ) on the output *y*, represented as percent changes. Note that in this example, a positive (in blue) or negative (in red) change in parameters can cause an increase or decrease in *y*.

The simplest sensitivity analysis uses input-output simulations, where parameters are increased and decreased one at a time to study their effects. The outputs that are important physiologically or included in the model calibration step should be investigated (23, 149, 150). The input-output relationship can provide a coarse approximation of the model sensitivity to the inputs. These results are typically portrayed using a “tornado plot” (151), as shown in Fig. 3. The plot can be read as follows: parameters in the model (θ_1_ … θ_5_) are increased (blue, +Δθ) and decreased (red, –Δθ) one at a time. The relative change in the output, *y* = *M*(θ), is then reported for each change. In the example shown in Fig. 3, increasing θ_1_ by 10% raised *y* by ∼60%, while decreasing θ_1_ by 10% reduced *y* by roughly 20%.

A more rigorous sensitivity approach is to perform a local sensitivity analysis, where sensitivity is described by derivatives. For simpler models, these sensitivities may be derived analytically; however, these sensitivities are often approximated numerically, e.g., using finite differences (152). Like identifying the slope of a curve, the local sensitivity of an output will only be valid in a small area around the initial value and provide limited information if the model exhibits numerous nonlinearities (152, 153). The most comprehensive method is global sensitivity analysis. These methods, which include the “distributed evaluation of local sensitivity analysis” (DELSA) method (11), the derivative-based global sensitivity measures (DGSMs) (12), Morris’ Screening (154), and Sobol’ indexes (155), are more computationally expensive than local methods. However, they provide more detailed information about model behavior and the effects of parameters. A summary of several global sensitivity methods and their application to cardiovascular models can be found in (156). Another common method outside the scope of this article is automatic differentiation, which has been used for sensitivity analysis in cardiovascular modeling studies (150) and is a fundamental component of current machine learning and AI architectures (157).

In summary, sensitivity analysis determines which parameters are the most influential on a given model output. At a minimum, we recommend using local sensitivity methods to identify whether the model outputs, especially those that appear in the cost function or likelihood, are sensitive to the parameter inputs. As discussed in the next section, sensitivity analyses provide quantitative justification for certain experimental designs that best inform the computational model.

### Identifiability

Parameters with no impact on the model output (i.e., that are noninfluential) are hard or impossible to identify during model calibration. The concept of identifiability relates to whether parameters can be estimated (or “fit”) based on the model structure or available data. Only systems with identifiable parameters can be used to correlate model inputs with biological measurements, as nonunique values lead to false conclusions about the underlying hypothesis. These topics are covered in detail in the recent review by Wieland et al. (158) in the context of quantitative systems biology, Sher et al. (159) for ionic and cellular electrophysiology models, and Miao et al. (27) for models of viral dynamics.

**Figure 4.**
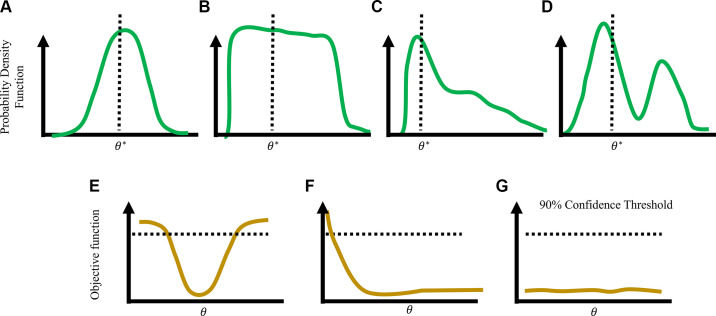
Posterior densities (*A–D*) and profile likelihood (*E*–*G*) results. *A*: example of a unimodal posterior distribution from an identifiable parameter. *B–D*: examples of posterior profiles that suggest either “difficult to identify” or nonidentifiable parameters. *E*: profile likelihood plot showing a unique minimum below the 90% confidence threshold for the parameter, suggesting practical identifiability. *F*: one-sided profile likelihood, indicative of a practically nonidentifiable parameter. More data will likely address identifiability issues. *G*: nonidentifiable parameter. It is likely this parameter is structurally nonidentifiable in the model.

Briefly, identifiability is either structural or practical. The former is related to model structure (e.g., are parameters directly multiplied together) whereas the latter is related to the data available for calibration. Structural identifiability can be addressed using available software packages (160) and has been applied to Windkessel and cardiac muscle kinetics models (28). Practical identifiability can be attributed to limited or noisy data (e.g., a low signal-to-noise ratio) and can be improved with additional experimental data or a new experimental design. Techniques like sensitivity analysis (23, 148), global optimization (29, 92, 142, 149), and posteriors obtained from Bayesian inference (128, 144, 161) can provide insight into whether parameters are practically identifiable. A local sensitivity, using derivative-based methods, can identify local parameter interactions and provide evidence for strong parameter correlations through an approximation of the parameter covariance matrix (23, 27). Posterior distributions from Bayesian inference that are nearly uniform, multimodal, or exhibit long tails can indicate practical identifiability issues. The most robust method is the profile likelihood (162, 163, 232) which provides confidence intervals for parameters given a fixed experimental design. Profile likelihood confidence intervals that are below a specified statistical significance threshold are considered nonidentifiable. Examples of posterior densities and profile likelihood confidence intervals are shown in Fig. 4. Further details are provided in appendix c.

Identifiability analysis is an underused but important step in the model workflow (as in Fig. 1). Detecting which parameters are identifiable can also inform which experimental designs are necessary and informative. Ideally, scientists performing modeling and experimental studies can synergistically assess which experiments maximally inform an in silico model. We recommend that investigators assess identifiability, provide evidence for whether model parameters were uniquely determined, and avoid interpreting parameter estimates that cannot be uniquely determined from available data.

### Model Calibration

After selecting a computational model, formulating the statistical problem, and determining influential and identifiable parameters, the model can be calibrated to the measured data. This is typically called parameter estimation or parameter inference. Scientists should be clear whether they are inferring a population-level parameter from pooled data [e.g., from a cohort of patients (164)] or if they are considering patient-specific parameters [e.g., calibrating to a single instance of clinical data (149)]. This is an especially important distinction in cancer medicine, as reviewed in detail by Brady and Enderling (165).

Deterministic (nonrandom) methods, such as gradient-based optimization, are readily available in software such as MATLAB (Natick, MA), Python, or the Java-based simulation tool JSim (166). These algorithms require an initial parameter guess (i.e., an a priori parameter set) and subsequently “search” the parameter space to reduce the cost function or maximize the likelihood. There are also evolutionary algorithms, such as particle swarm optimization and the genetic algorithm, which implement “evolution” strategies that update multiple parameters’ estimates to find the best global estimates (167).

Calculating the approximate posterior distribution in Bayesian inference requires sufficient coverage of the possible parameter values. Typically, Markov chain Monte Carlo (MCMC) sampling (168) is used. These methods draw samples from the posterior using the prior and likelihoods defined earlier. Common MCMC methods include the Metropolis-Hastings algorithm, Gibbs sampling, the adaptive Metropolis algorithm, and Hamiltonian Monte Carlo (169, 170). These methods use a sampling scheme to approximately sample parameters from the posterior distribution. The Metropolis-Hastings and adaptive Metropolis algorithms approximately sample from a proposal distribution, specified by the user, in which parameter values are either accepted or rejected if they improve (or only marginally decrease) the product of the prior probability odds and likelihood ratio. Hamiltonian Monte Carlo innovates on the prior two methods by using the gradient of the log likelihood and log prior. Variational inference is an alternative optimization technique in Bayesian statistics where a family of model distributions is compared with the unknown true posterior distribution. The mathematical details are beyond the scope of this article, but for more details and a more rigorous mathematical treatment, we recommend Chapter 4 in the textbook by Murphy (171). Several packages available for MCMC include *bayestestR* in R (172), *mcmcstat* in MATLAB (173), and *pymcmcstat* in Python (174).

The multitude of available software and algorithms makes model calibration extremely flexible. However, this also means that investigators must carefully choose an algorithm and, to some extent, justify this choice. Gradient-based optimization is commonly used in cardiovascular modeling, yet Bayesian inference (specifically MCMC methods) is also increasingly used now. Given that these two methods can provide different results during model calibration, we recommend that investigators clearly identify their methods, specifically optimization criteria and other miscellaneous tuning variables necessary, for model calibration and parameter inference.

### Model Validation

Model calibration uses experimental and clinical data to estimate model parameters. Additional data not used for calibration can be used as validation data. Model validation assesses whether the model simulations agree with measured data in the absence of formal calibration. As described in detail by Carusi et al. (30), model validation can be quantitative or qualitative. The former can be seen in the study by Estrada et al. (175), who constructed a multiscale model of the LV that included growth and remodeling due to cell-signaling cascades. The authors stimulated the experimental conditions from multiple studies and validated their model framework against measured LV mass over time. An alternative approach to validation can include sensitivity analysis. If a biophysical parameter is influential on a certain output, and this is supported by hypotheses and data, then the model retains this feature of the true biological system. Like validation in experiments, it is often difficult to quantify the degree of validation for a particular model (32). Nevertheless, validation and the use of noncalibration data as a check of the computational model’s capabilities are necessary components of the modeling workflow and should continue to be used when applying models to cardiovascular research. We recommend, when possible, that authors separate data into calibration and validation data, and consider model validation within their study design.

### Model Selection and Reduction

Most of the analysis steps mentioned above address issues with noninfluential or nonidentifiable parameters. In contrast, information criteria and model selection justify whether the number of free, inferred parameters should be reduced. Information criteria, such as the Akaike Information Criteria (AIC) or the Bayesian information criteria (BIC), weigh the quality of fit (i.e., the likelihood value) and model flexibility (i.e., the number of free parameters) to prevent overfitting. For example, if increasing the number of free parameters only improves the likelihood slightly, the AIC or BIC will suggest the original model. However, both AIC and BIC are based on asymptotic analyses and can be biased toward models with a high number of parameters. Hence, other information criteria including Deviance information criteria (DIC) and Watanabe-Akaike information criteria (WAIC) should also be considered. Gelman et al. (176) provide more cutting-edge model selection criteria as well, including marginal likelihood and Bayes factors.

Authors have used these criteria to compare *1*) different mechanistic models and *2*) similar mechanistic models but with different free parameters (177, 178). We recommend, at a minimum, that researchers use AIC or BIC to determine whether model complexity should be reduced for their problem. We also encourage using multiple information criteria, since these different methods could be biased toward certain model types.

### Measurement Error and Model Discrepancy

**Figure 5.**
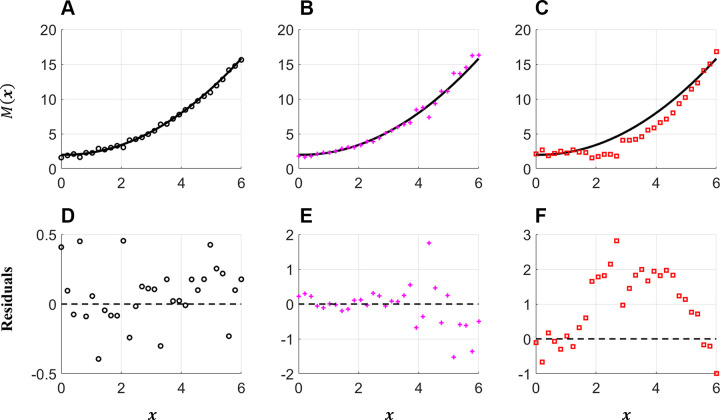
Test function (solid line) and various measurement error models (markers). The true model, *M*(*x*) = 0.4*x*^2^ − 0.1*x* + 2, is plotted along with Gaussian independent and identically distributed (iid) errors (*A*), Gaussian heteroskedastic errors (*B*), and correlated errors (*C*). *D–F*: the residuals. The iid errors (*D*) are random and have equal variance for all values of *x*. *E*: independent but heteroskedastic errors, where the errors are random, but the variance increases with *x*. *F*: correlated errors, identified by the nonrandom shape (positive for 2 < *x* < 5, negative otherwise) indicative of error correlation.

Typically, measurement errors are assumed to be independent and identically distributed (iid), meaning that the errors, ε, are independent of the values of *y*. A usual assumption is that measurement errors are iid from a normal distribution, with a mean of zero and some variance σ^2^, as shown in Fig. 5, *A* and *D*. A quantile-quantile (QQ) plot shows whether the residuals satisfy the assumption of normally distributed errors or if they are skewed left or right. When using multiple data sources or measurements collected across multiple temporal or spatial points, errors may be heteroskedastic, i.e., the variance of the measurement error may change with location or measurement device. Residuals from a heteroskedastic signal will still be independent and random, but the magnitude of the residuals will grow or shrink along the input dimension, as shown in Fig. 5, *B* and *E*.

Measurement error independence can be assessed by plotting the residuals after model calibration. Independent errors will exhibit a random pattern (Fig. 5*D*), whereas correlated or nonindependent errors will show an explicit pattern, as displayed in Fig. 5, *B*, *C*, *E*, and *F*. Correcting this mismatch requires changing the error model to have non-iid assumptions (e.g., additive or correlated noise) or accounting for model discrepancy. The former can be circumvented using established statistical techniques for regression and nonlinear regression models (179). The latter correction involves accounting for model discrepancy, the inability of the model to match the data due to missing physical or biophysical features (180). The studies by Lei et al. (181) and Paun et al. (182) provide examples of how to account for model discrepancy in cardiovascular applications.

Investigators should be aware of the potential pitfalls in their model analyses due to these (possibly wrong) statistical and model assumptions. A relatively new approach for identifying missing physics is equation learning (183, 184), which falls within the context of machine learning, which may be a useful avenue for addressing this issue. Model discrepancy is typically ignored in most cardiovascular modeling studies; however, we recommend that authors report if their residuals satisfy the iid assumption. If not, they should use heteroskedastic or correlated error models, or consider if a different mechanistic model or model discrepancy is needed in the calibration process.

## INPUT AND OUTPUT UNCERTAINTY

Uncertainty is an unavoidable aspect of scientific research. From an experimental perspective, we try to account for “user uncertainty’ by repeating measurements multiple times or assessing “sample uncertainty” by using multiple experimental trials or cell/animal subjects. A similar level of care must be taken when considering mathematical models of the cardiovascular system. Here, we define common terms related to mathematical and simulation uncertainty, specify available mathematical tools, and provide recommendations on the appropriate analyses to perform when using in silico models for analysis.

### Definitions

There are several terms used in the computational modeling community related to uncertainty. We recommend following the same definitions used by the ASME and found in the text by Smith (152):

- Verification: investigating the computational model for correct mathematical formulations, and further identifying numerical and discretization errors in approximating the true solution of the model;
- Validation: determining how accurate the model is in representing (bio)physical processes, with both experimental errors and model assumptions in mind; and
- Uncertainty quantification: numerical identification of uncertainties in input variables, parameters, and measurements and their effects on the variability of model outputs.

Model verification is less burdensome for ODE models, where time step size is the major contributor to numerical accuracy. However, individuals using PDE models (e.g., CFD, solid mechanics, or FSI models) need to address both temporal and spatial accuracies in their numerical scheme. Model validation is introduced in model calibration and includes qualitative or quantitative agreements between model simulations and observations from the physical system. Uncertainty quantification is covered further here.

### Data and Measurement Uncertainty

Measurement errors, as introduced and discussed at length in *Measurement Error and Model Discrepancy*, are typically assumed to be iid and normally distributed, with mean zero and constant variance. When matching to multiple data sources, the variance of each measurement modality (e.g., echocardiography vs. magnetic resonance imaging for cardiac volume) may also vary. As discussed in *Quantifying Model-Data Agreement* and *Model Calibration*, measurements with more uncertainty should be weighed less during model calibration. Error correlation, if suspected, should also be considered, as illustrated in Fig. 5, *C* and *F*. At a minimum, investigators should quantify or identify each data source’s uncertainty and whether one measurement modality is more reliable or consistent than the other.

### Parameter Uncertainty

Measurement errors affect model parameter estimates, and these uncertainties can be analyzed in either the frequentist or Bayesian frameworks, as introduced in *Frequentist and Bayesian Statistics*. In the frequentist framework, parameter confidence intervals are calculated based on the measurement error assumptions. For OLS with iid, normally distributed errors, the confidence intervals for an estimated parameter ${\hat{\theta }}_{i}$ are approximated using the standard error with *n* – *p* degrees of freedom, where *n* is the number of data points used for fitting and *p* is the number of parameters estimated, like linear regression (152). A similar approach can be used in nonlinear regression, which is appropriate for the nonlinear mechanistic models used in cardiovascular modeling. However, the standard error calculation for nonlinear regression has to be calculated based on the model sensitivity and can require significant computation time [see Smith (152) and Seber and Wild (185) for more details]. Nonlinear regression confidence intervals can be calculated using *nls* in R and MATLAB’s *nlinparci*. Again, note that a confidence interval in the frequentist framework indicates the probability that repeated experiments will contain the true, fixed value. In Bayesian inference, the posterior distribution will naturally give the uncertainty in the parameter estimates as well. A straightforward way to quantify the parameter uncertainty is to calculate the variance of a parameter’s marginal posterior distribution. appendix d provides theoretical details regarding parameter confidence intervals using nonlinear models.

Given the current requirements by multiple organizations and journals, including the American physiological society (186) and *AJP-Heart and Circ* (187), on reporting parameters and confidence intervals for statistical models, we encourage reporting mechanistic model parameters and their uncertainty (using either the frequentist or Bayesian approach) after calibration. Reporting these additional metrics of uncertainty complements the goal of improved rigor and reproducibility in publishing. Moreover, this practice may encourage more scientists to use mechanistic models in their design and analysis of physiological systems.

### Output Uncertainty

The uncertainties in the model inputs and data do not completely capture the uncertainties in the model outputs. Thus an additional step in the model analysis pipeline requires “propagating” the input uncertainties through the model. Quantifying output uncertainty is fundamental for using computational models to develop biomedical devices, as suggested by the ASME V&V 40 (188). The most basic approach for accounting for this uncertainty is to show the variability in model output when changing parameter values, one at a time, by a specified amount (similar to the sensitivity analysis and tornado plot in Fig. 2).

A more robust representation of output uncertainty requires revisiting the statistical model employed. This approach enables the calculation of confidence and prediction intervals for the model response (see appendix e for more details). Bayesian credible and prediction intervals can also be generated for model outputs. Credible intervals require reevaluating the computational model at a specified percentile of the posterior, and then identifying the pointwise mean and variance in the model outputs. Thus Bayesian output uncertainty quantification requires repeated evaluations from the model again after constructing the posterior. It should be noted that output uncertainty for expensive models (e.g., PDEs) can be expedited using “surrogate” models or “emulators.” The two most common approaches include polynomial chaos expansions (156, 189) and Gaussian processes (137, 190). Both are commonly used in other engineering disciplines and are gaining traction in the cardiovascular modeling domain.

Output uncertainty should be provided in any simulation study. The reason for this is twofold. First, there is some inherent variability or noise in measured data regardless of the measurement modality. Hence, even with a perfect model, noisy data can lead to biased calibration or simulation parameters. The second reason is that a majority of computational models include subsystems or outputs that do not have data for calibration or validation, e.g., the study by Colebank and Chesler (128) calibrated a model of biventricular interaction to different simulated experimental designs used to study the RV. The authors showed the calibrated model had substantial uncertainty in other simulated outputs (e.g., septal wall motion) that were not part of the calibration step. Thus the conclusions about a model after calibration can be biased to the data they are calibrated to, and uncertainty in the model output can help identify these biases.

We recommend performing at least one of these above techniques when using mechanistic models to study cardiovascular function. As mentioned in *Sensitivity Analysis*, we recommend that investigators evaluate how model outputs are affected by changes in the a priori or optimal parameter values. This initial step in quantifying output uncertainty should be complemented by more formal statistical methods.

## MODEL ACCESSIBILITY AND REPRODUCIBILITY

The ability to access and reproduce findings is the driving mechanism for scientific discovery. In line with previous paper calls from *AJP-Heart and Circ*, computational modeling should adhere to rigor and reproducibility standards. Public availability of the code for in silico models is similar to antibody reporting in in vitro and in vivo work, where authors must now include company names, lot numbers, and antibody dilutions in their publications. Here, we provide a summary of practices that will improve the accessibility, interpretability, and reproducibility of mechanistic computational models.

### Open-Source Software

The first step in making models accessible is to freely provide some, if not all, of the computational code. Hosting tools such as GitHub and GitLab provide users with the ability to efficiently work on code with collaborators and switch their repositories from “private” to “public” upon submission or acceptance of a study. Posting code after publication makes it easier for other scientists to use established models in the future. Notably, articles with publicly available data sets are associated with high citation counts (191), and we anticipate that articles including open-source code will trend similarly. There are also several licensing options that can be included in these repositories to ensure appropriate code use (192).

One potential limitation in using the above repositories is that they do not come with a robust identifier, such as a digital object identifier (DOI). To circumvent this, services like Zenodo and Figshare can provide a DOI for repositories, zip files, and other source code at the time of paper submission or publication. Attributing a DOI to code is especially important in the age of article preprint servers such as bioRxiv and medRxiv and will protect codes if they are made public in conjunction with a preprint. Another limitation is proprietary data or sensitive identifiers within clinical data. These issues are being tackled by funding agencies like the National Institutes of Health, which have adopted data management policies that are becoming common for data sharing.

We also emphasize that if authors decide to provide code and software “upon reasonable request,” then they must be held accountable for providing software to the inquiry. Thus we recommend that authors share a majority of their code when posting to public access sites, especially those files that recreate the figures within their published manuscript. In addition, we also recommend that authors submitting to *AJP-Heart and Circ* ensure that their code is available for reviewers during the review process and provide author contact information that will ensure the models are available years after publication.

### Modeling Languages and Packages

There are now multiple languages available for constructing computational models. Whereas the early cardiovascular models were primarily written in C, C++, and FORTRAN, recently developed models are now written in MATLAB, Java, Python, and Julia. In addition to software languages, there are several packages that are either partially or wholly focused on simulating cardiovascular function. ANSYS FLUENT (193), COMSOL (194), and Abaqus (41, 195) are all commercial software packages that can be used to simulate cardiovascular function using standard graphical user interfaces (GUI). There are also multiple open-source software packages that can be used for building computational models of the cardiovascular system. For CFD, FSI, and hemodynamic simulations, both SimVascular (55, 196) and the CRIMSON (56, 108) software are specifically designed to conduct image-based hemodynamics modeling. These software packages can handle 3D finite-element simulations, as well as other reduced order or lower fidelity CFD models. Several of the authors of the present manuscript have also published a MATLAB and C++ interface for running the 1D hemodynamics model originally proposed by Olufsen et al. (58, 143, 182, 197). For solid mechanics and cardiac modeling, the Cancer, Heart, And Soft Tissue Environment (CHASTE) (198, 199), the Open Continuum Mechanics, Imaging, Signal processing, and System identification (OpenCMISS) environment (200), and Continuity (201) are available and can be integrated with patient imaging data. More recently, the open-source software FEBio (202) has become a default simulation tool for cardiovascular solid mechanics. The increasing use of python has also led to several models using finite element packages, including FEniCS and others (36). The open Cardiac Arrhythmia Research Package (openCARP) for electrophysiology is one example of how languages like python are being used to advance the field of cardiovascular computation (203). A review of different cardiac solid mechanics models and their benchmark testing can be found in Ref. 36.

Software for cell or multiscale modeling also exists. CellML is an XML-formatted software for conducting single and multicell simulations (4). An entire repository of mathematical models available in CellML can be found at the Physiome Project website, including circulation, excitation-contraction coupling, metabolism, and myofilament mechanics models (6). For cardiac muscle and cross-bridge kinetics, the FiberSim (7) software as well as the open-source model developed by Marzban et al. (64) are available in MATLAB. Finally, there are several multiscale or systems-level modeling packages available. Two of the most popular are the HumMod modeling environment (8) and the CircAdapt model (16, 110). Both models include features of muscle mechanics, cardiac contraction, vascular hemodynamics, and cardiovascular adaptation or autonomic control. There are also more “noncoding friendly” software and packages available for those with little experience writing code. This includes MATLAB’s SIMULINK (21) and more recently the Modelica open-source software (204, 205), which use a “drag and drop” methodology to help visually construct complex, multicomponent models. Many of these software packages, including HumMod, CircAdapt, and Modelica, enable users to adapt the simulation framework for a specific problem of interest.

Researchers should also be aware of tradeoffs in model fidelity (e.g., spatially averaged flow simulations vs. spatially explicit flow simulations) and model computation time. For instance, 3D blood flow models can take anywhere from hours to days for a single simulation. In contrast, lumped parameter and systems-level models typically run in seconds to minutes. The tradeoff is most apparent in the detail of model resolution: the more complex and expensive the model, the more refined the spatial or temporal resolution of the simulation. An example is 3D CFD, which provides detailed spatial variation in wall shear stress. There are also presimulation requirements for each modeling infrastructure. Models that require patient-specific domains through imaging data will require image analysis (e.g., segmentation) and processing of the patient geometry (e.g., determining a 3D mesh of the heart). These preprocessing steps are nontrivial and contribute significant “offline” time before carrying out model simulations. However, much of the existing software now leverage statistical and machine learning techniques to assist in image segmentation and analysis (206). In addition, these same techniques are being used to speed up computation by replacing expensive hemodynamic simulators with robust, efficient emulators or surrogate models (207, 208).

## EXAMPLE WORKFLOW

We now provide an example model with in vivo data to highlight aspects of the workflow presented in this Guidelines article. We do not use every methodology mentioned here but use the minimal criteria suggested in the workflow.

First, we motivate the problem and introduce the modeling framework. Then, we identify the inputs and outputs of the model, the model parameters, and which outputs are used for calibration. We conduct a local sensitivity analysis to determine which parameters are most influential. We calibrate the model using the frequentist and Bayesian approaches to illustrate the differences between the two frameworks for model calibration. For the frequentist approach, we use global optimization, which also determines whether the model parameters are uniquely identifiable. In the Bayesian approach, we used the adaptive metropolis algorithm (209). We construct parameter confidence intervals (frequentist) and compare them to the marginal posterior distributions in the Bayesian approach. Finally, we produce confidence and prediction intervals for the model output in the frequentist framework, as well as credible and prediction intervals from the Bayesian framework. The model and analyses are conducted in MATLAB, and the software is available at https://github.com/mjcolebank/AJP_Guidelines.

### Experimental Data

The data used herein are from the study by Colebank et al. (26). Briefly, the data include LV pressure and volume signals, as well as systemic arterial pressure data, from an adult C57/B16 male mouse (20–22 wk old). All animal procedures were approved by the University of Wisconsin-Madison Institutional Animal Care and Use. Pressure and volume measurements were recorded at 500 Hz and analyzed on commercially available software (Notocord Systems, Croissy Sur Seine, France). Fifty sequential heartbeats were selected from the data and processed using MATLAB software, and the average pressure and volume signals were used for analysis.

### Computational Model

**Figure 6.**
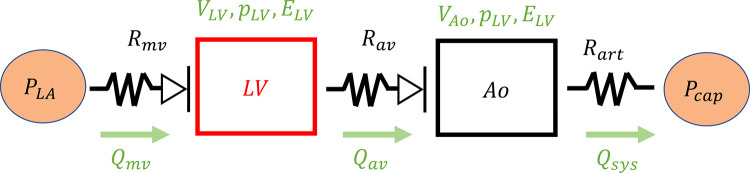
Model schematic. The model includes constant left atrial and systemic capillary pressures (P_LA_ and P_cap_) attached to a linear elastance model of the left ventricle (LV) and a compliant compartment model of the aorta (Ao). The resistors between the atrium and LV (*R*_mv_) and between the LV and Ao (*R*_av_) are modeled as diodes to mimic one-way flow across the valves. *R*_art_, arterial resistance. Green variables are time-dependent quantities in the model, including volume (V), pressure (P), elastance (*E*), flow across the mitral valve (*Q*_mv_), flow across the aortic valve (*Q*_av_), and flow from the aorta to the systemic capillaries (*Q*_sys_).

We use a lumped parameter model to describe the dynamics of the LV and aorta (corresponding to the systemic arterial pressure data). The model includes a time-varying, linear-elastance model for the LV pressure-volume relationship and an electrical circuit analog for the aorta. The LV is preceded by a constant left atrial pressure source, and the aortic compartment is attached to a constant systemic capillary pressure source. Resistance elements are located between the left atrium, LV, and aorta. The first two are diodes, mimicking one-way valves, while the last resistance element represents the resistance of the small arteries and arterioles. A schematic of the model is provided in Fig. 6. Model equations can be found in appendix f. We assume that the constant pressure sources for the left atrium and systemic capillaries are P_LA_ = 5 mmHg and P_cap_ = 20 mmHg.

The quantities of interest for the sensitivity analysis and model calibration are LV volume, LV pressure, and aortic pressure. We compare dynamic pressure and volume predictions over the cardiac cycle to the measured time-series data. An example hypothesis (given a larger population of data from both sexes) would be that model parameters describing end-systolic elastance and aortic stiffness will be significantly different between female and male mice after calibrating to measured data (210). The model parameters are:

(*7*) $$\theta =\left\{R_{\text{mv}},R_{\text{av}},R_{\text{art}},E_{\text{es}},E_{\text{ed}},T_{\text{s}},T_{\text{e}},V_{\text{LV},\text{d}},C_{\text{ao}}\right\}$$

which includes the resistors, *R* (mmHg·s/μL), capacitors representing compliance, *C* (μL/mmHg), elastance, *E_i_* (mmHg/μL), timing variables *T_i_* (s), and dead volume V_LV,d_ (μL). The independent variable is time, *t*, while the dependent variables are the volumes, flows, and pressures between the left atrium and the systemic capillaries. We assume that the cardiac cycle length is constant and set to 0.11 s (26).

### A Priori Parameter Values and Sensitivity Analysis

The nine parameters in *Eq. 7* are given a priori values based on the measured data and physiological relationships. Valve resistances (*R*_mv_, *R*_av_) are set to relatively small values since there is no sign of valvular disease, and *R*_art_ is approximated by the assumed pressure difference between aortic and capillary pressure divided by the cardiac output (see Table 2). End-systolic and end-diastolic pressure and volume values are used to obtain initial elastance values (*E*_es_, *E*_ed_). Timing parameters are initialized with maximal ventricular pressure development (*T*_s_) and the start of diastole (*T*_e_). The dead volume in the LV (V_LV,d_) is set at 5 μL, and the aortic compliance (*C*_ao_) is set as the ratio of stroke volume and systemic arterial pulse pressure (164).

**Table 2.** Summary of the model parameters, their interpretation, and/or formula for calculation and the optimal values and confidence intervals for estimated parameters

Parameter	Physiological Interpretation	Formula	A Priori Value	Parameter Range	Optimal Value	Confidence Interval
P_LA_, mmHg	Left atrial pressure		5			
P_cap_, mmHg	Systemic capillary pressure		20			
*R*_mv_, mmHg·s/μL	Mitral valve resistance		5e-3	[1e-4, 1e-2]	4.41e-3	[2.52e-3, 6.29e-3]
*R*_av_, mmHg·s/μL	Aortic valve resistance		1e-3			
*R*_art_, mmHg·s/μL	Arterial resistance	$\frac{\left({\bar{P}}_{\text{Ao}}-{\text{P}}_{\text{cap}}\right)}{\text{Cardic output}}$	2.68e-1	[0.1, 1.0]	3.29e-1	[2.96e-1, 3.62 e-1]
*E*_es_, mmHg/μL	End-systolic elastance	$\frac{\text{max}\;\left({\text{P}}_{\text{LV}}\right)}{\text{min}\;\left({\text{V}}_{\text{LV}}\right)}$	4.85	[0.5, 6.0]	4.42	[4.21, 4.64]
*E*_ed_, mmHg/μL	End-diastolic elastance	$\frac{\text{min}\;\left({\text{P}}_{\text{LV}}\right)}{\text{max}\;\left({\text{V}}_{\text{LV}}\right)}$	1.46e-1	[1e-2, 1.0]	1.49e-1	[1.20e-1, 1.79e-1]
*T*_s_, s	Time of maximum elastance		4.40e-2	[0.33, 0.66]	3.92e-2	[3.79e-1, 4.04e-1]
*T*_e_, s	Time of minimum elastance		7.70e-2	[0.66, 0.88]	8.22e-2	[8.09e-2, 8.36e-2]
V_LV,d_, μL	Dead volume in left ventricle		5	[1, 10]		
*C*_Ao_, μL/mmHg	Aortic compliance	$\frac{\left[\text{max}\;\left({\text{V}}_{\text{LV}}\right)-\text{min}\;\left({\text{V}}_{\text{LV}}\right)\right]}{\left[\text{max}\;\left({\text{P}}_{\text{Ao}}\right)-\text{min}\;\left({\text{P}}_{\text{Ao}}\right)\right]}$	9.68e-1	[0.4, 1.5]	5.48e-1	[4.71e-1, 6.24e-1]

Parameter range is included for any parameters used during global optimization, whereas optimal values and confidence intervals are reported for the 7-parameter subset discussed in main text. P_Ao_, aortic pressure; P_LV_, left ventricular pressure; V_LV_, left ventricular volume.

**Figure 7.**
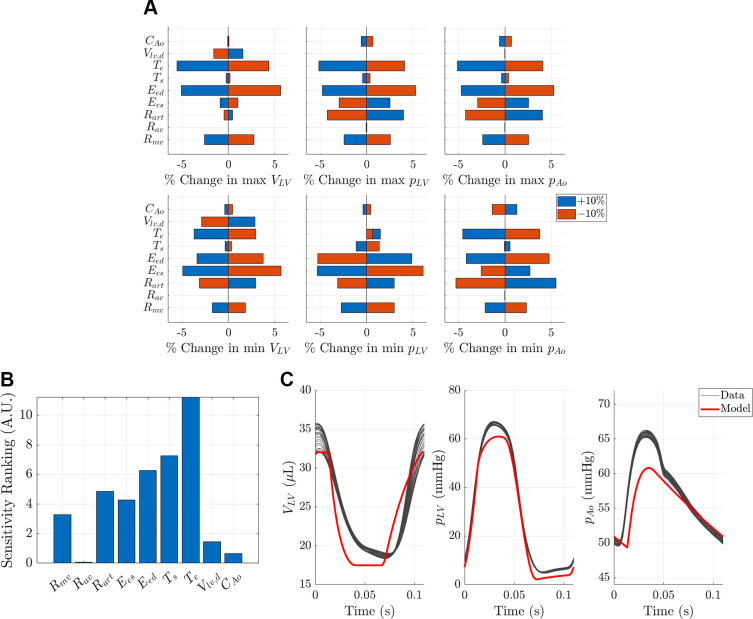
Model analysis. *A*: initial sensitivity analysis and tornado plot show how parameters affect maximum and minimum values of simulated left ventricular (LV) volume (V_LV_), LV pressure (P_LV_), and aortic (Ao) pressure (P_Ao_). *B*: a more formal local sensitivity analysis using all 3 time-series predictions as the quantity of interest helps identify which parameters are collectively important on the 3 simulated outputs [in arbitrary units (A.U.)]. In general, *R*_av_ is noninfluential and can be fixed at its a priori value. *C*: a priori simulations agree somewhat well with the data across 50 heartbeats. *C*_Ao_, aortice compliance; *E*_es_, end-systolic elastance; *E*_ed_, end-diastolic elastance; *R*_art_, arterial resistance; *R*_av_, aortic valve resistance; *R*_mv_, mitral valve resistance; *T*_e_, time of minimum elastance; *T*_s_, time of maximum elastance; V_LV,d_, dead volume in left ventricle.

We use sensitivity analysis to identify which parameters are most influential on the model outputs. First, we examine how a 10% change in parameter values affects the systolic and diastolic values, as shown in the tornado plot in Fig. 7*A*. In general, every parameter, except the aortic valve resistance *R_av_*, influences the model. Then, we use a local, derivative-based sensitivity analysis with the LV volume, LV pressure, and aortic pressure time-series vectors as our quantities of interest. Since these outputs have different units and magnitudes, we scale each output by the average value of the respective data source. The local sensitivities are converted to a scalar ranking metric by calculating the sum of squared sensitivities, ∑*S_i_*(*t*)^2^, where *S_i_*(*t*) is the sensitivity of the outputs to each parameter *i*.

The local sensitivities shown in Fig. 7*B* suggest that *R*_av_ is noninfluential and can be fixed at its a priori value. The parameters *T*_e_, *T*_s_, *E*_ed_, *E*_es_, and *R*_art_ are the most influential parameters. We use a correlation analysis to identify parameters that show any pairwise interdependence on the outputs and find that *E*_es_ and V_LV,d_ have a strong (0.97) correlation. We fix V_LV,d_ = 5 given this correlation and its relatively small effect on the simulated outputs. This leaves seven parameters for model calibration. Figure 7*C* shows the model simulations against the data using the a priori parameter values. Table 2 provides the a priori formulas.

### Frequentist Model Calibration

The model is relatively inexpensive computationally (roughly 1 s per forward solution). Thus we initialize a global optimization for the parameters {*R*_mv_, *R*_art_, *E*_es_, *E*_ed_, *T*_s_, *T*_e_, *C*_ao_} by randomly selecting 100 initial values and running optimizations using the cost function:

(*8*) $$J\left(\theta \right)=\sum_{i=1}^{N}{\left({\text{D}}_{i}^{\text{V},\text{LV}}-{\text{V}}_{\text{LV}}\left(t_{i};\theta \right)\right)}^{2}+\sum_{i=1}^{N}{\left({\text{D}}_{i}^{\text{p},\text{LV}}-p_{\text{LV}}\left(t_{i};\theta \right)\right)}^{2}+\sum_{i=1}^{N}{\left({\text{D}}_{i}^{\text{p},\text{Ao}}-p_{\text{Ao}}\left(t_{i};\theta \right)\right)}^{2}.$$

This cost function simultaneously minimizes the differences between the LV volume data (${\text{D}}_{i}^{\text{V},\text{LV}}$), LV pressure data (${\text{D}}_{i}^{\text{p},\text{LV}}$), and aortic pressure data (${\text{D}}_{i}^{\text{p},\text{Ao}}$) and their corresponding simulations. Note that, since the model outputs are similar in magnitude, we do not scale or weigh the three data sources.

A global optimization serves as both an identifiability investigation and a calibration step. There is a global minimum from the optimization, with a specific region of parameter space corresponding to the minimum in the objective function. While not all 100 initial guesses lead to the optimal parameter vector (i.e., some get stuck in local minima), the 20 parameter sets corresponding to the 20 smallest calibration errors are nearly identical. These 20 estimates have a coefficient of variation (the standard deviation relative to the mean) ≤3% for all seven parameters, suggesting a global minimum. In contrast, running optimization with V_LV,d_ included led to a high coefficient of variation (27%), suggesting identifiability issues.

We then investigated whether a smaller subset of estimated parameters was comparable in quality of fit. The sensitivity analysis results suggest that *C*_ao_, *R*_mv_, and *E*_es_ are the least influential parameters of the subset. We systematically reran our global optimization to determine if leaving these parameters fixed at their a priori value influenced the quality of fit. We compare the models using the AIC score, where the smaller the value, the better the model. The original parameter subset {*R*_mv_, *R*_art_, *E*_es_, *E*_ed_, *T*_s_, *T*_e_, *C*_ao_} has the best AIC score (≈ 627) compared with the subsets without *R_mv_* (AIC ≈ 629), without *C*_ao_ (AIC ≈ 879), and without *E*_es_ (AIC ≈ 677). Hence, the seven-parameter subset is sufficient and necessary for calibration.

### Bayesian Model Calibration

To illustrate the differences between the two statistical methodologies, we also performed Bayesian model calibration using MCMC. We use the adaptive metropolis algorithm, which is detailed in full in the text by Haario et al. (209), using the *mcmcstat* MATLAB package (available at https://mjlaine.github.io/mcmcstat/). The cost function shown in *Eq. 8* is adapted to a likelihood function. We assume uniform prior distributions for each parameter over the parameter ranges provided in Table 2. We run our MCMC analysis using 100,000 iterations.

**Figure 8.**
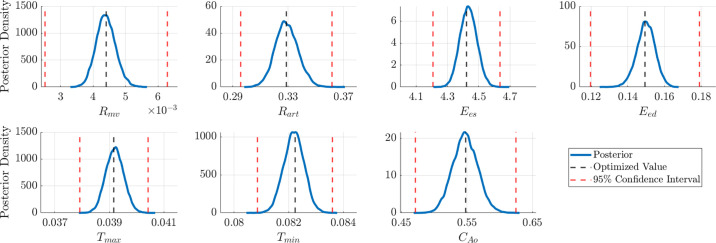
Parameter uncertainty. Optimal parameter values determined from 100 initializations of optimization are provided in black, along with the frequentist 95% confidence intervals in red. Marginal parameter posteriors are provided in blue. Note that the marginal posteriors are unimodal with the optimal parameter estimate from the frequentist optimization coinciding close to the posterior mode. *C*_Ao_, aortic compliance; *E*_es_, end-systolic elastance; *E*_ed_, end-diastolic elastance; *R*_av_, aortic valve resistance; *R*_mv_, mitral valve resistance; *T*_e_, time of minimum elastance; *T*_s_, time of maximum elastance.

The marginal posterior distributions for the parameters are provided in Fig. 8. The marginal posteriors are unimodal in shape and appear normally distributed, which is due in part to the uniform prior distribution. Our findings again suggest that our parameters are practically identifiable given the relatively tight posterior distributions. We also show the global minimum from our frequentist optimization result in Fig. 8, which is closely aligned with the mode of the parameter posteriors.

### Uncertainty Quantification

**Figure 9.**
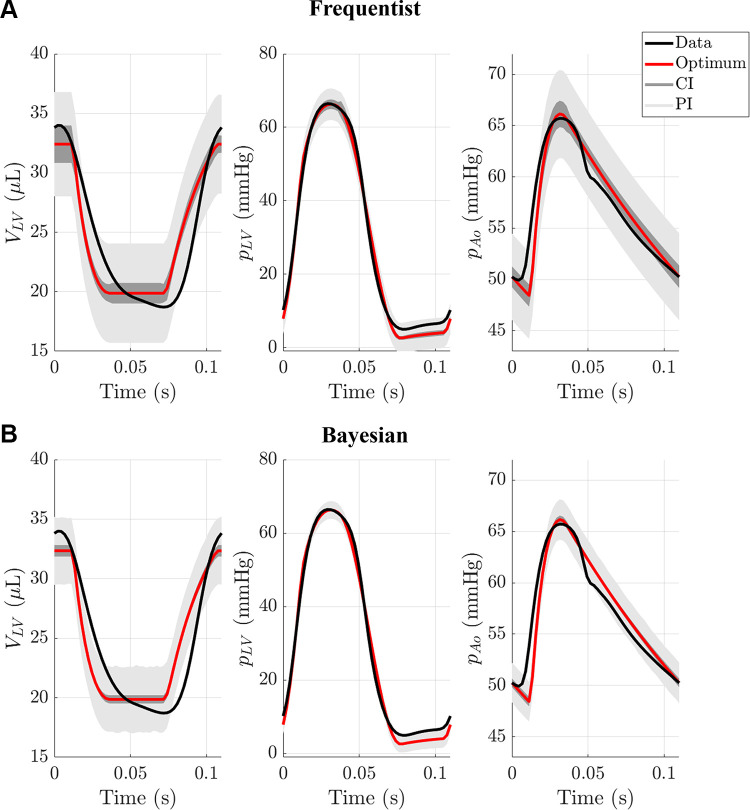
Output uncertainty after performing either frequentist (*A*) or Bayesian (*B*) model calibration. Frequentist uncertainty included 95% confidence (CI) and 95% prediction intervals (PI). Optimum agrees well with the pressure (P) data in left ventricle (P_LV_) and aorta (P_Ao_), whereas there are more apparent discrepancies between the left ventricular (LV) volume (V_LV_). In the Bayesian setting, uncertainty is described by 95% credible and prediction intervals. The uncertainty is smaller in the Bayesian setting, although the prediction at the posterior model (shown in red) is nearly identical to the frequentist result.

Finally, we construct frequentist parameter confidence intervals and output uncertainty for both frequentist and Bayesian methods. We use the built-in MATLAB functions *nlpredci* and *nlparci* for the output and parameter uncertainty analysis, respectively, in the frequentist framework. The parameter confidence intervals are plotted with the marginal posterior distributions from the Bayesian inference in Fig. 8 and are also provided in Table 2. Optimal model simulations and their respective confidence and prediction intervals are provided in Fig. 9. For Bayesian output uncertainty, we draw 2,000 samples from the marginal posterior (as well as the noise variance estimate) to construct 95% credible and prediction intervals.

The frequentist parameter confidence intervals for *R*_mv_ and *E*_ed_ are the largest intervals (relative to their magnitude), while the timing parameters have the tightest relative confidence bounds (43% and 20% of the mean vs. 2–3% of the mean, respectively). This also holds for the Bayesian framework, with frequentist and Bayesian methods overlapping substantially in Fig. 8. The output uncertainty in Fig. 9*A* shows a relatively larger confidence interval in volume output than the LV and aortic pressure, which is related to the model sensitivity (which is larger relative to the pressure outputs). Note that the prediction intervals, which account for noise in newly acquired data, are wider than the confidence intervals and contain the true signals. The Bayesian credible intervals in output space shown in Fig. 9*B* are noticeably smaller than the frequentist confidence intervals, which is attributed to the difference in approach (i.e., frequentist uses an approximate model sensitivity, Bayesian uses posterior samples). However, the Bayesian prediction intervals are closer in magnitude to the frequentist prediction intervals and show larger relative uncertainty in LV volume output. In general, both models capture a similar fit to the measured data.

Finally, the residuals for both frequentist and Bayesian results do not appear to be iid, suggesting that there is either error correlation or, more likely, model discrepancy due to missing physics in the model. We acknowledge that this is a limitation of the present model, and, although outside the scope of the present manuscript, recommend either using a more advanced statistical model to handle these errors or increasing the mechanistic model complexity (i.e., using a more sophisticated LV and aortic model).

## SUMMARY, FUTURE PERSPECTIVES, AND CONCLUSIONS

We have provided a basic review of cardiovascular modeling as well as fundamental methods for model development, calibration, and analysis. Taking these factors into account, we propose a standardized workflow for the development and use of mechanistic, computational, in silico models in the context of cardiovascular function. Table 1 and Fig. 1 summarize the recommended steps and workflow for the start and end of the model analysis pipeline.

The future of cardiovascular innovation relies on a combination of experimental investigations, data analysis, and, as noted here, computational modeling. The push toward “precision medicine,” “patient-specific treatments,” and “medical digital twins” is being driven by data integration across multiple modalities and spatial or temporal scales. The availability of large databases has led to multiple scientific discoveries using machine learning and, more broadly, artificial intelligence (AI). Now, scientists without traditional computational training are advancing patient care in the clinic through these techniques (211). While machine learning is an active field of research, it is limited in that *1*) it is driven by measurement observations, which can be sparse or noisy and *2*) it typically lacks interpretability (212), although this latter point is being actively addressed. We believe that these tools should be used in combination with mechanistic models, as machine learning and mechanistic modeling can complement each other to expedite scientific discovery. An example of this includes the work by Jones et al. (113) who used machine learning in combination with lumped parameter, systems-level models to identify distinct heart failure phenotypes.

Computational models are an effective tool for integrating multimodal data and constructing patient-specific simulators for physiological investigations (213). Toward this end, computational models are now considered “devices” for clinical diagnostics. This has led to several initiatives by regulatory agencies, such as the FDA, to establish modeling and simulation standards. The FDA has highlighted the importance of in silico clinical trials and product testing as a potential innovation in the medical device community. The limiting factor of these models, however, is credibility and lack of detailed standards for model evaluation (214). Consistent with several points that we have highlighted in this Guidelines article, a list of “Ten Rules of credible practice of modeling and simulation in healthcare,” (1) was recently published by the Interagency Modeling and Analysis Group (IMAG), a working group representing, the National Institutes of Health, National Science Foundation, FDA, and other federal agencies.

The development of robust, reproducible models also depends on data availability. The editors of *AJP-Heart and Circ* have highlighted the lack of experimental studies that consider sex as a biological variable. In response, *AJP-Heart and Circ* published over 60 articles focused on sex differences in cardiovascular function (215). This initiative is imperative to the computational modeling field, as the scarcity of data has translated to biased model calibration and development of “male” models of cardiovascular function. We expect that computational modeling will serve as an additional tool in detecting sex differences in various cardiovascular diseases and may lead to new innovations in model components that account for sex, such as hormone contributions (216, 217), the lack of sex hormones (218), and the effects of pregnancy (109, 175).

In a similar vein, there is an urgent need to identify and study markers of health disparities. Several *AJP-Heart and Circ* articles have highlighted differences in vascular function (219), cardiac vagal activity (220), and cardiac DNA methylation (221) across racial groups. This limited area of data availability again limits the broad applicability of computational models, as most data sets used for model calibration and development are not inclusive in their participant cohort or do consider patient demographics. The use of modeling as a tool requires awareness of the possible model limitations due to data bias. Modeling may provide new insight into health disparities, as the ability to integrate data from multiple sources and generate novel hypotheses with computational modeling could help identify and motivate studies into experimental blind spots. Modeling and simulation must also abide by ethical considerations. Models that identify gene expression and signaling pathways (85) or require electronic health records (124, 149) should be aware of how collections of data used for patient-specific modeling run the risk of becoming identifiable. In addition, studies that exploit computational modeling to identify mechanisms of disease that are unique across patient demographics should be aware of biases and incorrect conclusions. Modeling and simulation should advance healthcare for all individuals, and models should be developed with the intention of improving public health.

Finally, there are several computational modeling technologies and concepts that are paving the way for the future of cardiovascular medicine. The mass availability of data and increased computational power of modern computers has elevated machine learning and data assimilation methods. This includes automated cardiac and vascular image segmentation (222, 223), high-dimensional data analysis in heart failure (83), and even machine learning-informed surrogate models, including models constructed from 3D CFD simulations in coronary artery lesions (224). Machine learning methods can also be integrated with the techniques mentioned here to improve patient-specific outcomes, such as combining patient-specific parameter estimates with unsupervised machine learning methods (113). While machine learning and AI methods are outside the scope of this Guidelines article, a similar standard of model documentation, analysis, and availability is recommended for these methods. Modeling and simulation practices have already integrated AI techniques (208). These methods are ideal for learning relationships between measurables, whereas mechanistic modeling builds mechanistic relationships between hypothesized subsystems of human physiology. A combined approach, where machine learning is used to help identify missing biophysics (225) or identify relationships in multiscale models, is ideal so that the mechanisms that explain relationships in the measured data are interpretable and translatable. The fundamental ideas provided here regarding uncertainty are also paramount when using AI with in silico models and should be considered, especially in the case of data that are both high dimensional in the number of features and suffer from measurement errors.

A promising direction in cardiovascular research is the development of digital twins of the heart and cardiovascular system (226, 227). Like patient-specific models, digital twins are a virtual, online simulator of human physiology that is routinely updated with new data from either *1*) sequential follow-up visits or *2*) remote sensor technologies. This developing area borrows ideas from mechanistic modeling, machine learning, and statistical inference fields, together allowing for the ability to monitor a patient’s physiology across short and large time scales. A combination of the techniques listed here, as well as FDA guidelines on models and medical devices, will be required to move the field of digital twins forward.

In summary, we provide a list of practices and techniques that assist in identifying appropriate computational models, their limitations, and their strengths. We suggest these guidelines to promote both scientific rigor and reproducibility. Computational models are useful tools for connecting data across multiple experimental scales, measurement modalities, and organ systems to study cardiovascular function. Their use should be steered by credible guidelines and practices, as highlighted here.

## GRANTS

M.J.C. was supported through National Institutes of Health (NIH) Grant TL1-TR001415. C.M.W. was funded by the National Science Foundation CMMI Grant 2030173 and American Heart Association and Children’s Heart Foundation Grant 20CDA35210754. D.A.B. was funded by NIH Grant HL139813. D.A.B. and N.C.C. were funded by the NIH Grant R01-HL154624. M.S.O. and N.C.C. were funded by NIH Grant R01-HL147590. D.H. was supported by United Kingdom Engineering and Physical Sciences Research Council Grant EP/T017899/1.

## DISCLAIMERS

The content is solely the responsibility of the authors and does not necessarily represent the official views of the NIH.

## DISCLOSURES

No conflicts of interest, financial or otherwise, are declared by the authors.

## AUTHOR CONTRIBUTIONS

M.J.C. conceived and designed research; M.J.C. performed experiments; M.J.C. analyzed data; M.J.C. interpreted results of experiments; M.J.C. prepared figures; M.J.C. and N.C.C. drafted manuscript; M.J.C., P.A.O., C.M.W., A.G., D.A.B., D.H., M.S.O., and N.C.C. edited and revised manuscript; M.J.C., P.A.O., C.M.W., A.G., D.A.B., D.H., M.S.O., and N.C.C. approved final version of manuscript.

## APPENDIX A

### Bayesian Statistics

Briefly, the notation *P*(*A* | *B*) can be interpreted as “the probability that B is true, conditioned on A being true;” i.e., we are interested in the probability of B being true after observing A. Let *y* represent the measured data, θ the parameters of the system, and *M*(θ) the model of the physical system. Bayes’ theorem requires four terms. The first component is the prior distribution, *P*(θ | *M*), discussed above, which is conditioned on the model framework. The second term is the likelihood function, *P*(*y*|θ, *M*), which expresses how likely or probable the data are conditioned on our current knowledge of the parameters and how well our model simulation, *M*(θ), aligns with the data. The likelihood increases when the model and data are aligned, which also means that the distance between the data and the model simulation decreases (explained in detail in *Quantifying Model-Data Agreement*). The third term is the evidence or marginal likelihood, P(y), which represents the sum over all values of priors and likelihood. The final term is the posterior distribution, *P*(θ | *y*, *M*), which expresses the probability (and uncertainty) of the parameter values conditioned on the data and the model. The posterior distribution reflects how the prior information, model framework, and available data reflect our belief and confidence in values that a parameter can take on. Bayes’ rule is then written as:

(*A1*) $$P\left(\theta |y,M\right)=\frac{P\left(y|\theta ,M\right)P\left(\theta |M\right)}{P\left(y\right)}.$$

*Equation 4* can be read as “the probability of the parameters, conditioned on the model and data, is proportional to the likelihood of the data and the prior beliefs around the parameter value.” It can be noted that the evidence, *P*(*y*), in the denominator also serves as a “normalization constant,” such that the probabilities integrate to 1.

## APPENDIX B

### Cost Functionals, Likelihoods, And Maximum Likelihood

Problems that deal with multiple data sources require a modified objective function. A more general objective function to handle different measurements is:

(*B1*) $$J=\sum_{d=1}^{D}\sum_{i=1}^{N_{d}}w_{d,i}{\left(y_{d,i}-M_{d,i}\left(\theta \right)\right)}^{2},$$

where the double sum is over the number of experimental outputs, *D*, and the number of measurement points for that output, *N_d_*. In this more realistic setting, only some of the specific model outputs *M_d_*_,_*_i_*(θ) are compared with the various experimental measurements. The additional variables *w_d_*,*_i_* represent the “weights” prescribed to each experimental output. These can be selected to improve fits to certain experimental data or can be specified to reflect the uncertainty in the measurements (i.e., smaller weights for more noisy data).

The likelihood function, as introduced in model calibration, increases (i.e., becomes “more likely”) as the model data agreement improves. Assuming that the measurement noise is Gaussian and iid, the likelihood function is expressed as:

(*B2*) $$L\left(y|\theta \right)=\frac{1}{\sqrt{2\pi \sigma^{2}}}\exp\;\left(\frac{-1}{2\sigma^{2}}\sum_{i=1}^{N}{\left(y_{i}-M_{i}\left(\theta \right)\right)}^{2}\right),$$

where σ^2^ is the noise variance as defined previously. For certain noise distributions, such as the Gaussian, the maximum likelihood estimate is equivalent to minimizing the cost function for ordinary least squares in *Eq. 6*. In the case of multiple measurement modalities, authors should use multivariate Gaussian noise to represent multiple measurement sources that still satisfy the iid assumption. In this setting, the likelihood is written as:

(*B3*) $$L\left(y|\theta \right)={\left(2\pi \right)}^{-\frac{N}{2}}{\left(\det\;\left(\Sigma \right)\right)}^{-\frac{1}{2}}\;\exp\;\left(-\frac{1}{2}{\left(y-M\left(\theta \right)\right)}^{⊤}\Sigma^{-1}\left(y-M\left(\theta \right)\right)\right)$$

where the covariance matrix *Σ* is a diagonal matrix (size *N* × *N*) with the individual noise variances, σ^2^, on the diagonal. When the iid assumption is not valid (i.e., when there is error correlation), Σ will have off-diagonal elements that represent the error correlation between data points.

The maximum likelihood estimator has three optimality guarantees that distinguish it from other estimators: asymptotic unbiasedness, consistency, and asymptotic efficiency. To understand these criteria, we revisit the estimation framework discussed in model calibration. Frequentist parameter estimation is based on the concept of repeated measurements (data), where each measurement or sample from the population is subject to a different noise instantiation. For each of these data, we get a different parameter estimate, and we can compute its mean and its variance. Intuitively, an estimator is “good” if the mean is close to the unknown true parameters, if the estimate converges to the true parameter as the data set size increases, and if the uncertainty, quantified by the variance, is as small as possible. This is effectively what the three-optimality criteria guarantee. For a methodologically more rigorous discussion, see Chapter 4 in the text by Murphy (171). The likelihood approaches formulated in Appendix *Eqs. B2*, *B3*, and *D1* provide these theoretical optimality conditions, whereas ad hoc methods for model calibration can eliminate any theoretical conclusions.

## APPENDIX C

### Identifiability Analyses

Identifiability is split into two different subgroups. Model parameters that cannot be uniquely determined because of model structure (e.g., 2 parameters multiplied together) are considered structurally nonidentifiable, and the models themselves are sometimes considered structurally nonidentifiable models (158). In this setting, even an infinite amount of data would not remedy the issue of nonuniqueness in the parameter estimates. In contrast, practical identifiability assesses whether the parameters can be uniquely inferred from limited, noisy data. Throughout this section, we focus on the concept of practical identifiability (see Ref. 158 for more details).

The most feasible local identifiability method is analyzing the local sensitivity matrix (23, 148). If two parameters provide identical sensitivities, they likely have similar effects on the model and might not be uniquely identified during calibration. The local sensitivity can also be used to construct a correlation matrix (148). Although correlation does not indicate identifiability, several authors have shown that nearly perfect correlations between outputs (e.g., ±0.97–1.00 correlation coefficients) lead to nonidentifiable parameter subsets (124, 148, 228). More robust, global identifiability methods also exist. The simplest approach is to randomly perturb the parameters in a system and then use optimization to identify global and/or local minima. Global optimization methods include the genetic algorithm, particle swarm optimization, and multistart optimization (229). Several cardiovascular studies (29, 92, 142, 149) have used these techniques to either determine a global minimum for parameter estimation or to test for identifiability. In the context of Bayesian statistics, several authors have assessed practical identifiability using MCMC techniques (128, 144, 161). These methods use the marginal posterior densities to assess identifiability. Nonidentifiable or weakly identifiable parameters may appear uniform, have multiple modes, or have long posterior tails. When dealing with multiple parameters, posterior correlations between parameters can assess the uniqueness of each individual parameter. A schematic representation of these posteriors is shown in Fig. 4, *A–D*.

Finally, one of the most robust practical identifiability tools is the profile likelihood (162, 163). This method “profiles” a single parameter by fixing one parameter at a time and inferring all other parameters simultaneously and leads to a more precise construction of parameter confidence intervals. While robust, this method is computationally expensive and typically only feasible for more simplistic and efficient computational models. Parameters that have confidence intervals with finite bounds (e.g., a quadratic profile likelihood) are considered identifiable; parameters that have at least one infinite bound for their confidence interval are considered nonidentifiable (158). The shape of the parameter profile can be further interrogated: parameters with a flat profile likelihood are typically structurally nonidentifiable, whereas a parameter that is only bounded above or below is thought to be practically nonidentifiable and could be remedied with additional data. We illustrate these profiles in Fig. 4, *E–G*. This approach has been applied several times to lumped parameter models of the cardiovascular system (128, 230, 231), as well as models of ion channels and action potentials (87, 88).

## APPENDIX D

### Parameter Uncertainty

For OLS with iid, normally distributed errors, the confidence intervals for an estimated parameter ${\hat{\theta }}_{i}$ are approximated by:

(*D1*) $${\hat{\theta }}_{i}\pm {\text{t}}_{N-\text{p}}^{1-\alpha /2}\sqrt{V_{\mathrm{ii}}},V=\sigma^{2}{\left(\chi^{⊤}\chi \right)}^{-1}$$

where $t_{N-p}^{1-\alpha /2}$ is a two-sided *t* value corresponding to a (1 – α)% confidence interval with *N* – *p* degrees of freedom. Here *N* is the number of data points used for calibration, and *p* is the number of parameters estimated. The confidence intervals also depend on the parameter covariance estimator matrix, *V*, which is constructed from the error variance estimate, σ^2^, and the design matrix χ. In linear regression, the design matrix, χ = *X*, contains covariates corresponding to measurement instances. In nonlinear regression, the covariance matrix can be approximated by the local sensitivity, *∂M* / *∂θ_i_*, as discussed earlier, but at the value of the estimated parameters, $\hat{\theta }$. More details are found in the texts by Smith (152) and by Seber and Wild (185). We again note that a confidence interval in the frequentist framework does not indicate the probability of a parameter taking on a value but instead indicates the probability that repeated experiments will contain the true, fixed value. Moreover, complex nonlinear models may not be well approximated through sensitivity analyses, leading to underestimated or incorrect confidence intervals (158).

In Bayesian inference, parameter uncertainty is incorporated into both the prior and posterior distribution. The marginal posterior distributions for the parameters are dependent on the prior parameter distributions and their uncertainty, as well as the ability of the model to match the data (i.e., the likelihood). A key meaning of this statement is that the choice of prior will have an impact on the posterior uncertainty for the parameters (152). With this choice in mind, the widths of the posterior parameter densities are representative of the parameter uncertainty conditioned on the prior and the likelihood.

## APPENDIX E

### Output Uncertainty

Frequentist output confidence and prediction intervals are obtained in a similar way to those for parameter confidence intervals. With the optimal parameter estimate, $\hat{\theta }$, corresponding to the optimal model output, $M\left(\hat{\theta }\right)$, and assuming iid, normally distributed measurement errors, the model confidence intervals are:

(*E1*) $$\left[M_{\text{CI}}^{-},M_{\text{CI}}^{+}\right]=M\left(\hat{\theta }\right)\pm {\text{t}}_{N-\text{p}}^{1-\alpha /2}\sqrt{x_{0}^{⊤}\text{V}x_{0}},$$

where $x_{0}^{⊤}\approx dM\left(\hat{\theta }\right)/d\hat{\theta }$ requires the local sensitivity of the model at the optimal parameter value, $\hat{\theta }$, and *V* is the covariance estimator defined in Appendix *Eq. D1*). Prediction intervals for the model response look nearly identical but also incorporate the noise variance, giving:

(*E2*) $$\left[M_{\text{PI}}^{-},M_{PI}^{+}\right]=M\left(\hat{\theta }\right)\pm {\text{t}}_{N-\text{p}}^{1-\alpha /2}\sqrt{x_{0}^{⊤}\text{V}x_{0}+\sigma^{2}}.$$

The difference between the confidence and prediction interval is that the confidence interval accounts for uncertainty in the model output based on the measured data, whereas prediction intervals include the uncertainty in new measurements. Since the new measurements are subjected to measurement error, they are wider than the confidence intervals. Appendix *Eq. E2* can also be extended to account for error correlation or error heteroskedasticity (26). Output uncertainty in the Bayesian framework can be computed by sampling from the approximate posterior and constructing credible intervals around the simulations. Predictions intervals can be produced in a similar way to the credible intervals, with the additionally uncertainty accounted for from the measurement error estimates (152).

## APPENDIX F

### Example Model

The example model used in the main text consists of a constant left atrial and pulmonary capillary pressure source, P_LA_ and P_cap_, respectively, connected to a time-varying elastance model of the LV and a linear compliance model of the aortic compartment. The change in volume in the LV and aortic compartment (V_LV_ and V_Ao_) are described through mass conservation:

(*F1*) $$\frac{{\text{dV}}_{\text{LV}}}{\text{d}t}=Q_{\text{mv}}-Q_{\text{av}},\frac{{\text{dV}}_{\text{Ao}}}{\text{d}t}=Q_{\text{av}}-Q_{\text{sys}}.$$

The change in volume is dictated by the flow across the mitral valve (*Q*_mv_, μL/s), the flow across the aortic valve (*Q*_av_, μL/s), and the flow from the aorta to the systemic capillaries (*Q*_sys_, μL/s). The flows are proportional to the ratio of the pressure gradient across the compartments and the resistance between compartments, giving:

(*F2*) $$Q_{\text{mv}}=\max\;\left(\frac{{\text{P}}_{\text{LA}}-{\text{p}}_{\text{LV}}}{R_{\text{mv}}},0\right),Q_{\text{av}}=\max\;\left(\frac{{\text{p}}_{\text{LV}}-{\text{p}}_{\text{Ao}}}{R_{\text{av}}},0\right),Q_{\text{sys}}=\frac{{\text{p}}_{\text{Ao}}-{\text{P}}_{\text{Sys}}}{R_{\text{sys}}}.$$

The valves are assumed to inhibit backflow; hence, *Q*_mv_ and *Q*_av_ are modeled as diodes to ensure only positive flow. Pressure in the LV and Ao are simulated using:

(*F3*) $${\text{p}}_{\text{LV}}=E\left(t\right)\left({\text{V}}_{\text{LV}}-{\text{V}}_{\text{d}}\right),{\text{p}}_{\text{Ao}}={\text{V}}_{\text{Ao}}/C_{\text{Ao}}.$$

where *E*(*t*) is the linear, piecewise elastance model described by:

(*F4*) $$E\left(t\right)=\{\begin{array}{c} \;\frac{\left(E_{\text{es}}-E_{\text{ed}}\right)}{2}\left(1-\cos\;\left(\frac{\pi t}{T_{\text{s}}}\right)\right)+E_{\text{ed}},0\leq t<T_{\text{s}}\\ \;\frac{\left(E_{\text{es}}-E_{\text{ed}}\right)}{2}\left(1+\cos\;\left(\frac{\pi \left(t-T_{\text{e}}\right)}{\left(T_{\text{e}}-T_{\text{s}}\right)}\right)\right)+E_{\text{ed}},T_{\text{s}}\leq t<T_{\text{e}}\\ \;E_{\text{ed}},\;T_{\text{e}}\leq t<T \end{array}$$

which is applied periodically across each heartbeat.
